# Stimuli-Responsive Phase Change Materials: Optical and Optoelectronic Applications

**DOI:** 10.3390/ma14123396

**Published:** 2021-06-19

**Authors:** Irene Vassalini, Ivano Alessandri, Domenico de Ceglia

**Affiliations:** 1 INSTM, Research Unit of Brescia, Via Branze 38, 25123 Brescia, Italy; ivano.alessandri@unibs.it; 2 Department of Information Engineering, University of Brescia, Via Branze 38, 25123 Brescia, Italy; 3 CNR-INO, Research Unit of Brescia, Via Branze 38, 25123 Brescia, Italy; 4 Department of Information Engineering, University of Padova, Via Gradenigo 6/a, 35131 Padova, Italy

**Keywords:** phase change materials, GST, VO_2_, thin films, metasurfaces, active photonics, displays, optical switches, memories

## Abstract

Stimuli-responsive materials offer a large variety of possibilities in fabrication of solid- state devices. Phase change materials (PCMs) undergo rapid and drastic changes of their optical properties upon switching from one crystallographic phase to another one. This peculiarity makes PCMs ideal candidates for a number of applications including sensors, active displays, photonic volatile and non-volatile memories for information storage and computer science and optoelectronic devices. This review analyzes different examples of PCMs, in particular germanium–antimonium tellurides and vanadium dioxide (VO_2_) and their applications in the above-mentioned fields, with a detailed discussion on potential, limitations and challenges.

## 1. Introduction

The optical properties of photonic devices are usually fixed at the time in which the devices are designed and then fabricated. A stimulating challenge in optics and photonics is to go beyond this static, passive behavior, and to gain some degree of control over the optical response, with the ultimate goal to achieve dynamically tunable, or even reconfigurable, optical functionalities [1,2]. The complication in achieving this goal derives from the fact that the most common materials used in photonics, such as glass for fiber optics and silicon for integrated photonics, hardly change their optical properties, even under intense optical or electrical stimuli. For this reason, optical modulation and switching, and more generally tunability, are usually reached by means of free-carrier injection, chemical and mechanical activation, or by deploying materials with large electro-optic or optical nonlinear coefficients [3,4,5,6,7]. All these mechanisms are inherently weak and require intense control signals and long interaction lengths in order to produce significant optical modulation. For this reason, researchers have devoted a great deal of attention to finding new, “smart” materials, in which the change of the refractive index can be large and, at the same time, accessible with relatively low-intensity stimuli.

In this context, Phase Change Materials (PCMs) represent a specific class of solid-state materials that undergo a transition from a given crystallographic phase to another one in response to an increase of temperature. This transition is combined with a significant variation of material optical and electrical properties [8,9]. They can be considered as a particular type of stimuli-responsive materials, since they modulate their electrical and optical behaviors in response to the application of an external stimulus, which leads to a variation of temperature. This stimulus can be delivered by direct heating, application of an electrical potential (the enhancement of temperature is a consequence of Joule heating) or laser pulses (laser heating). In particular, in response to the external stimulus PCMs pass from a phase characterized by low reflectivity, low refractive index, low dielectric optical constant and high resistivity (they behave as semiconductors) to a phase characterized by high reflectivity, high refractive index, high dielectric optical constant and low resistivity (they behave as metals) [1,10]. From this general description, it is evident that they can be employed in the preparation of photonic devices (that guide, manipulate and detect light) which are able to modify their function in response to external stimuli, behaving in a dynamic way. This possibility opens the way to the development of new competitive photonic systems, characterized by adjustable optical properties and on-demand functionalities. 

The main aim of this work is to analyze and discuss recent and selected examples that can be found in the literature of photonic systems obtained by using phase change materials which find applications in three particular fields: colored dynamic displays, optical switching and memory/logic devices. 

## 2. Types of Phase-Change Materials and Their Properties

**Figure 1:**
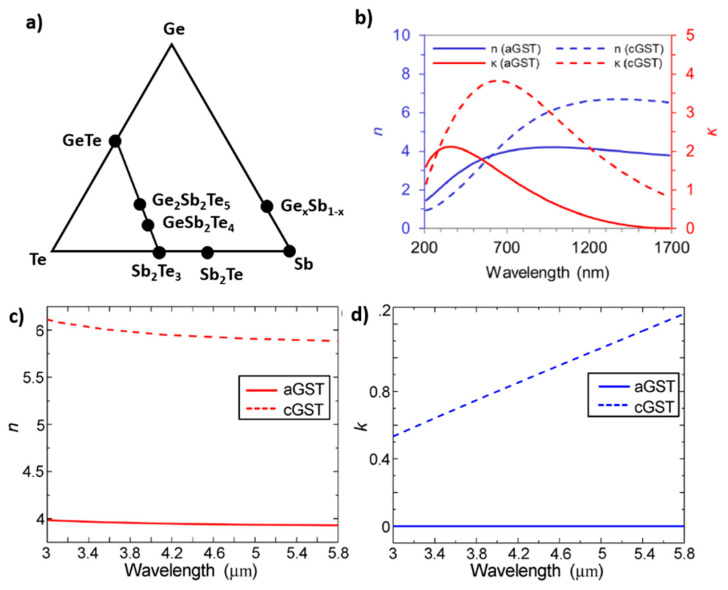
(**a**) Ternary phase diagram for Ge, Sb and Te showing most common chalcogenide phase change materials; (**b**) variation in the visible range of the real (*n*) and imaginary (*k*) part of the refractive index of Ge_2_Sb_2_Te_5_ (GST225) upon phase transition [14]; (**c**) variation in the IR range of the real (*n*) part of the refractive index of GST225 upon phase transition [15]; (**d**) variation in the IR range of the imaginary (*k*) part of the refractive index of GST225 upon phase transition [15]. Reproduced from Refs. [14,15] with permission from Optical Society and MDPI.

In general, in PCMs the two phases exhibit high contrast of the optical constants (Δ*n* > 1) over a broad wavelength range, from the visible to the infrared (IR) range [11]. The most studied and most frequently applied class of materials is that of chalcogenides, which are composites of elements of the XVI group of the periodic table (S, Se, Te) with Ge, As, Sb or Ga. In particular, the group of composites located on the GeTe–Sb_2_Te_3_ tie line in the ternary phase diagram for Ge, Sb and Te, such as GeSb_2_Te_4_ (GST124) and Ge_2_Sb_2_Te_5_ (GST225) (Figure 1a). Their phase change transition has been widely studied and it has been pointed out that their significant variation of electrical and optical properties is ascribed to a variety of types of bonds between the different atoms passing from amorphous (covalent bond) to crystalline phase (resonant bonding) [12]. The presence in the crystalline phase of resonant bonding (i.e., a single, half-filled p-band forms two bonds to the left and right (more than allowed by the 8-N rule)) has been identified as a unique fingerprint of this type of PCMs, that is obtained when the bond between the atoms in the crystalline phase are characterized by small ionicity and a limited degree of hybridization [13]. The materials are characterized by low reflectivity and low values of refractive index and extinction coefficient in the amorphous state, while the refractive index and extinction coefficient values significantly increase upon crystallization, leading to high reflectivity. In Figure 1b–d, the case of Ge_2_Sb_2_Te_5_ is reported as an example.

For this class of PCMs, crystallization occurs with heating in the range between 100 and 180 °C (with an increase of the transition temperature T_c_ moving from Sb_2_Te_3_ towards GeTe, and an increasing of T_c_ upon reducing the thickness of the material film or enhancing the residual stress to which it is subjected), while the melting point is around 600 °C (also, in this case, the glass transition temperature increases, moving from Sb_2_Te_3_ towards GeTe). Other interesting features are that the phase transition is very quick (in the order of ps-ns for amorphization and in the order of sub-nanoseconds and nanoseconds for crystallization) and not-volatile. The high rate of switching between the two different phases is justified by the fact that they are characterized by similar atomic arrangements [12]. For example, Ge_2_Sb_2_Te_5_ has an f.c.c structure when crystalline, which is characterized by high- grade lattice symmetry, similar to that of the amorphous state. Thus, upon crystallization, the atoms have to move only a little to achieve the right position inside the crystal structure. The key point to underscore is that the phase transition and related change of properties are permanent and non-volatile. This means that they are retained also after the removal of the inducing stimulus (i.e., temperature increase). This feature is highly suitable in many applications, because it enables us to create devices and functionalities that require a very low amount of energy to operate, just the one needed to induce the phase transition. Once crystallization is obtained, it is possible to revert the material back to the amorphous phase by heating it to a temperature higher than its melting point and making cooling happen quickly. In this case, it is possible to exploit both direct heating, or electric or laser pulses. In this case, after the removal of the external stimulus the chilling of the material has to be fast, so that the material atoms have no time to arrange themselves in the ordered crystalline structure. Switching between the amorphous and crystalline phases can be repeated various times, adjusting temperature properly. For example, different devices have been prepared with the capability of 10^5^ switching cycles for optical storage applications, or 10^6−17^ switching cycles for memory applications (10^6−8^ cycling capability is required for replacing flash memories, 10^12^ cycling capability is required for embedded memories and up to 10^15−17^ cycling capability is required for replacing standalone or embedded Dynamic RAM) [16,17].

Other materials included in this review are GeTe [18], Ge_2_Sb_2_Se_4_Te_1_ [19], Sb_2_Te_3_ [20], Ag_3_In_4_Sb_76_Te_17_ [21] and InSb [22]. All of them exhibit properties and responses that are similar to Ge_2_Sb_2_Te_5_.

**Figure 2:**
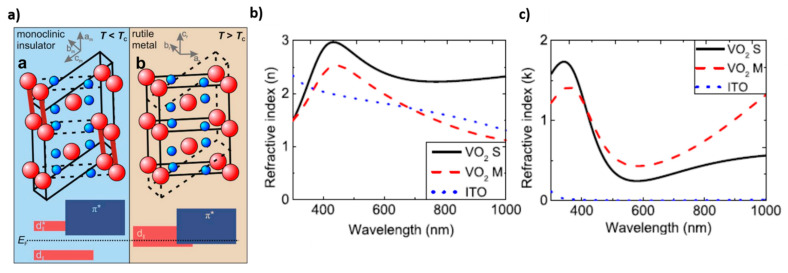
(**a**) Insulator to metal phase-change transition in VO_2_ [23]; (**b**) variation in the visible range of the real part of the refractive index of VO_2_ upon phase transition [24]; (**c**) variation in the visible range of the imaginary (*k*) part of the refractive index of VO_2_ upon phase transition [24]. Reproduced from Refs. [23,24] with permission from Nature Springer and IOP publishing.

On the other hand, special attention will be dedicated to vanadium oxide, VO_2_. Despite it is an oxide, it shares with the above-mentioned chalcogenide a crystallographic phase transition which leads to a significant change of refractive index and increases in conductivity. In this case, the material passes from monoclinic (insulating) to tetragonal (metallic) rutile structure (at T~68 °C). Above *T_c_*, the electrical resistance lowers by 3–5 order of magnitude and there is a dramatic change of the optical properties in the Vis-IR region (Figure 2). Below *T_c_*, VO_2_ is in the monoclinic phase and the V atoms pair with a high localization of d-electrons which leads to the formation of an energy gap of 0.6 eV, permitting high IR transmission. On the contrary, at temperatures higher than the transition temperature, there is an overlap between the Fermi level and the V 3d band which eliminates the aforementioned band gap, causing the material to be highly reflective or opaque in the near-infrared (NIR) region.

Unlike the GSTs and, more generally, chalcogenides, in VO_2_ the phase transition is volatile. This means that, once direct or indirect heating is stopped and temperature comes back to a value lower than transition temperature, the newly acquired properties are lost.

## 3. Variably Colored and Writable Displays

Phase change materials have been widely exploited for the preparation of both variably colored displays, where the color of the final device is determined by the morphological properties of the structure (such as PCM thickness, shape, spacing, phase, etc.), and writable displays, where the capability of the material of changing optical properties in response to external stimuli is exploited to store optical information (writings) with precise spatial and temporal control. In the following, different strategies will be illustrated according to the different types of involved materials. In general terms, the employment of PCMs represents an efficient alternative possibility to store optical information, which enriches the palette of strategies that can be found in the literature [25,26,27,28,29,30,31]. In particular, it entails some advantages: the possibility of achieving not only binary systems but multicolor devices with precise color depth modulation, the possibility of exploiting different stimuli (especially, laser and voltage), precise spatial control and very quick actuation. In addition, in the proposed devices, PCMs are especially in the form of thin films, requiring only a small amount of materials, and enabling the obtainment also of flexible devices.

### 3.1. Colored and Dynamic Displays Based on Interference Effect with Chalcogenides

**Figure 3:**
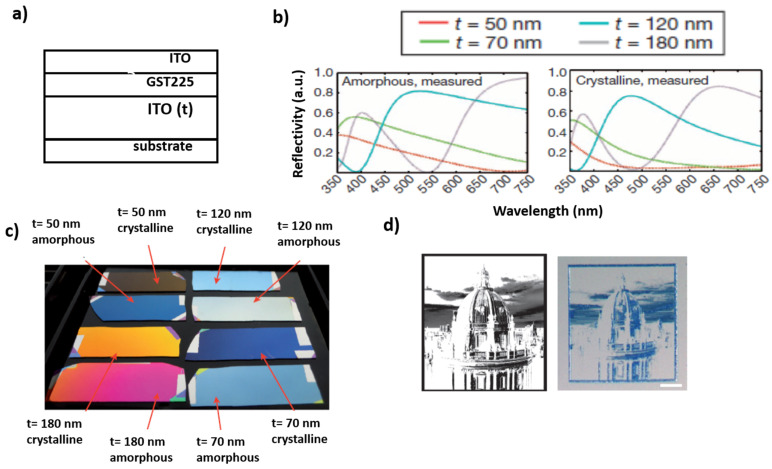
(**a**) Scheme of the structure proposed by Hosseini et al. [32] to obtain a colored display; (**b**) variation of the Reflectivity spectrum of a device consisting of 10 nm ITO/7 nm GST225/t ITO/Pt mirror, with different thickness t, upon phase transition; (**c**) optical pictures of different devices consisting of 10 nm ITO/7 nm GST225/t ITO/Pt mirror, with different t, in different phases; (**d**) employment of a 10 nm ITO/7 nm GST225/70 nm ITO/Pt mirror device for the reproduction and storage of a greyscale picture of the Oxford Radcliff Camera (original picture shown in the left). Clear regions correspond to amorphous GST225, dark blue regions correspond to crystalline GST225. The scale bar is 10 μm. Reproduced from Ref. [32] with permission from Nature Springer.

One of the first demonstrations of the use of a phase change material for the preparation of colored display that can be found in literature is the work of Hossein et al. [32], which is based on the employment of a Fabry–Pérot cavity made of two layers of Indium Tin Oxide (ITO), filled with Ge_2_Sb_2_Te_5_, which serves as a highly absorptive medium (Figure 3a). According to the type of substrate on which this stacked structure is deposited, it is possible to obtain a reflective (deposition on a refractive surface made of Pt) or a semi-transparent (deposition on a transparent material, such as quartz) display. Similarly, they demonstrated that it is possible to achieve both flexible (deposition on transparent boPET or Pt-covered boPET) and not-flexible devices. The final color of the display depends on the thickness of the bottom layer of ITO (since it determines the resonance condition of the whole cavity) and on the thickness of the Ge_2_Sb_2_Te_5_ layer. The same research group developed a theoretical model that enables us to simulate and predict the reflective spectrum of the multilayered system and convert it, by means of an algorithm, into the corresponding final color [33]. Thanks to these simulations it has been possible to determine the maximum thickness of the GST225 layer, which has to be lower than 25 nm, otherwise, the final display tends to a constant grey color, typical of bulk GST225. In addition, according to this model, it is possible to achieve a maximum value of contrast between the reflective spectrum of the whole system with GST225 in the amorphous or in the crystalline state, when the GST225 layer has a thickness lower than 10 nm. The most striking feature of the proposed system, in fact, is that by changing the crystallographic state of the GST225 layer it is possible to induce a variation of its refractive index, which is converted into a modification of the reflectivity (or transmission) spectrum of the whole stack, as visible in Figure 3b. Practically, this means a dynamic variation of the display color, in response to the phase change of the GST225 layer. For example, when the bottom ITO layer is 180-nm thick, and the GST225 layer has a thickness of 7 nm, the display is pink-purple if GST is in the amorphous state, and orange if GST is in the crystallin state, indicating a significant blueshift of the cavity resonance upon crystallization (Figure 3c). The authors demonstrated that it is possible to achieve this color modification by heating the whole structure or by applying a sufficient difference of potential (electrical stimulation) between the upper and the bottom layers of ITO thanks to the employment of a conductive tip. In particular, they created a pixelated display, in which each pixel can be independently accessed through the interaction with a conductive tip of an atomic force microscope (CAFM) in order to apply the sufficient difference of potential and make the GST225 phase transition occur. This strategy has been employed to reproduce photography in a bimodal color scale (Figure 3d).

The GST225 phase transition is not-volatile, leading to permanent storage of optical information, with reduced energy consumption. In fact, it is necessary just to supply to the system the energy necessary to cause the first (amorphous → crystalline) transition. Nevertheless, it is possible to erase the stored optical information on demand, by inducing a subsequent re-amorphization of the “drawn” pixel, thanks to the application of a higher-energy stimulus. The authors estimated that in the optimized display (with a very thin GST225 layer of 7 nm) the voltage threshold for the “writing” of one pixel is 2.2 V, while a pulse of 5 ns and 5 V is necessary for the erasing process (with a total energy consumption of only 15 pJ).

**Figure 4:**
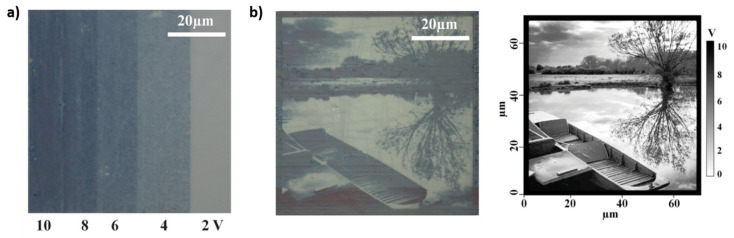
Non-binary grayscale image rendering. (**a**) different colors in reflection mode obtained by scanning voltage varying between 0 and 10 V and inducing different grades of crystallization by means of CAMS; (**b**) reproduction and storage of a greyscale picture of the boat picture shown in the left. The used device is a stacked 10 nm ITO/7 nm AIST/70 nm ITO/Pt sample. Reproduced from Ref. [21] with permission from Wiley.

The first possibility of achieving a not-binary color rendering of an image is reported by Rios et al. [21] The exploited system is analogous to the one just described, but in this case, the entity of the variation of color is linked to the amount of crystalized PCM in a certain CAFM scanned area. If the separation between two nuclei of crystallization is larger than the size they grow after the voltage pulse, some amorphous material remains in between, and the obtained final color is intermediate between those of the whole-amorphous and whole-crystalline state. Interestingly, they demonstrated that the nuclei size depends on the applied voltage, while the entity of separation relies on the scanning speed. So, by increasing the applied voltage (from 2 to 10 V) they were able to achieve a perceptible color depth modulation, which is linked to a different crystallization degree of the PCM layer (Figure 4a). In this case, the final color of the display dynamically depends on the crystallographic state of the PCM material, while statically depends on the thickness of both the PCM and the upper ITO reflective layer. In this work, the authors investigated as PCM both Ge_2_Sb_2_Te_5_ and Ag_3_In_4_Sb_76_Te_17_ (AIST). Figure 4b gives as an example the possibility of a storage a greyscale image on a 10 nm ITO/7 nm AIST/70 nm ITO with perceptible color depth modulation. Despite the two materials are characterized by different crystallization dynamics (GST225 undergoes nucleation dominated crystallization, while AIST a growth dominated crystallization) they enable us to obtain displays with very similar properties and performances, with a slightly better depth modulation capability and broader color gamut in the case of AIST. 

The possibility of achieving a multilevel optical tuning thanks to a gradual crystallization of the PCM layer inside a Fabry–Pérot cavity has been reported also by Meng et al. [34], who employed as PCM C-doped Sb_2_Te_3_, which is characterized by a very fast growth-dominated crystallization process, and, thanks to C-doping, by a continuous variation of crystallization degree as a function of temperature (not ON/OFF switching behavior). They demonstrated that by means of single-shot ultrafast (ps) laser pulses characterized by different laser fluence (14–32 mJ/cm^2^) it is possible to control the extent of the crystallized area and obtain 6 different reflectivity levels.

**Figure 5:**
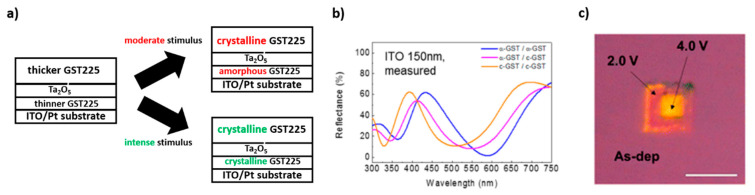
Examples of displays that can dynamically switch between more colors based on multilayered structures, which contain more than one layer made of a PCM. (**a**) Different PCM layers are characterized by different thicknesses and, as a consequence, different crystallization temperatures, so it is possible to achieve a selective crystallization of GST225 according to the intensity of the applied stimulus. Upon moderate heating or applied potential, only the thicker layer crystallizes, upon intense heating or applied potential both the GST225 layers crystallize; (**b**) measured reflectance spectra for 8 nm GST225/6 nm Ta_2_O_5_/5 nm GST225/150 nm ITO/Pt device upon different grades of stimulus and crystallization [35]; (**c**) optical microscope image (scale bar: 10 μm) of the phase and color transition induced by applying different potentials [35]. Reproduced from Ref. [35] with permission from the American Chemical Society.

Another strategy that can be employed to achieve multiple color switching is that of preparing multilayered structures, which contain more than one layer made of a PCM characterized by different thicknesses, separated by thermal/diffusion barriers. As anticipated in the previous paragraph, in the case of thin (<10 nm) film, the crystallization temperature of Ge_2_Sb_2_Te_5_ is significantly affected by thickness (T_c_ significantly increases with decreasing film thickness). For example, Yoo et al. proposed a multilayered structure composed of an 8 nm-thick GST225 layer, 6 nm-thick Ta_2_O_5_ thermal barrier layer, 5 nm-thick GST225 layer, ITO layer (whose thickness influences the final display colors) and Pt layer [35]. Since the upper GST225 layer is characterized by a major thickness, it crystalizes at a lower temperature than the underlying thinner GST225 layer. So, when the whole system is subjected to a moderate stimulus (annealing at 210 °C) only the thicker upper layer of GST225 undergoes the phase transition, while when the system is subjected to a more intense stimulus (annealing at 260 °C) both the GST225 layers crystallize (Figure 5a). Gradual crystallization leads to a progressive shift of the reflectance curves towards a shorter wavelength, enabling to obtain three different colors (Figure 5b). The authors theoretically demonstrated that, in general, it is possible to achieve a display that can switch between (*n* + 1) different colors, by stacking n PCM layers. The upper limit for n turns out to be 4. They demonstrated that it is possible to obtain color-switching also electrically, by applying different voltage biases: when a 2 V bias is imposed, only the thicker GST225 layer crystalizes, while at 4 V applied, both layers undergo a phase transition. The result is that three different colors can be dynamically visualized in the display (Figure 5c). 

Similarly, Jafary et al. recently proposed a reconfigurable color reflector by means of a selective phase transition in a multilayered structure obtained by staking GeTe layers of different thickness [36]. In this work, the employed PCM is GeTe (whose crystallization temperature varies according to the material thickness, similarly to GST225) and the employed stimulus for the occurrence of the phase transition and the color variation is Joule heating, by means of heaters integrated inside the structure. They demonstrated the appearance of four (2*n*) different colors, when two (*n*) layers of GeTe are involved: red when both GeTe layers are in the amorphous state, orange when only the bottom thinner layer is crystalline, green when only the upper thicker layer is crystalline and dark blue when both the layers are crystalline. 

A reconfigurable color reflector based on selective phase change in different layers has been proposed also by Meng et al., but by employing different phase change materials characterized by different phase change transition temperatures: GST225 (transition at ~160 °C) and VO_2_ (transition at ~70 °C) [37]. The optimized nanostructure is a multilayer device, in which the layers are deposited in the following order from the bottom to the top: SiO_2_/VO_2_ (70 nm)/GST225 (50 nm)/Al(100 nm). According to the phase-type of VO_2_ and GST225, four different reflectivity levels can be reached at four different temperatures, with a total reflectivity change of 44% over the whole visible spectrum. At room temperature, the structure is 87% reflective (this corresponds to the first reflective state); by heating the whole system up to 72 °C the reflectivity decreases to ~68%, indicating the metal-to-insulator transition of the VO_2_ layer, thus producing the second reflective state; by increasing furtherly the temperature up to 158 °C, the GST225 layer undergoes a structural phase transition, thus decreasing the overall reflectivity to the third reflective state of ~56%. Upon cooling from 200 °C to ambient temperature, a fourth reflective state of ~100% is achieved at 44 °C, which is due to the VO_2_ reversible transition back to an insulator behavior. Upon cooling, only one transition occurs, because the GST225 layer remains in its metastable, f.c.c. phase. 

Similar switchable and writable colored displays based on strong interference phenomenon are the systems reported by Schlich et al. based on a GST225 layer deposed on Au film and separated by a Ti/TiN layer, which acts as a diffusion barrier, and whose color switch has been obtained by means of fs-laser pulses [38], or the work by Shi et al., employing a GST225 thin layer deposited on a SiO_2_ dielectric layer and an Al mirror thick film [39].

### 3.2. Colored and Dynamic Displays Based on Structured Metasurfaces Containing Chalcogenides

Another strategy is based on the exploitation of analogous multilayered structures, where at least the upper layer is a structured metasurface. An example is the work of Gholipour et al., reporting a multilayered structure of subwavelength nanograting arrays made of ZnS/SiO_2_, GST225, ZnS/SiO_2_ deposed on a quartz substrate [40]. The presence of subwavelength period nanograting introduces resonances for TM-polarized light (which is incident perpendicularly to the grating lines) in the visible range. The precise value of the wavelengths of these resonances depends on the grating period, the line depth of the grating and the crystallographic state of the GST225 layer. When GST225 is in the amorphous state, resonances are due to displacement currents associated with the high refractive index contrast between dielectric GST225 and the surrounding medium; on the contrary, when GST225 is in the crystalline state, they are plasmonic resonances based upon the opposite sign of the real part of the relative permittivity at the interface between GST225 and the surrounding medium. Since different values of resonance wavelength mean different color, changing the grating period or the line depth enables to obtain a static variation of display color. In addition, the modification of the GST state enables to achieve a dynamic modulation of color display, in response to the application of an external stimulus, such as annealing or laser fs-pulses.

**Figure 6:**
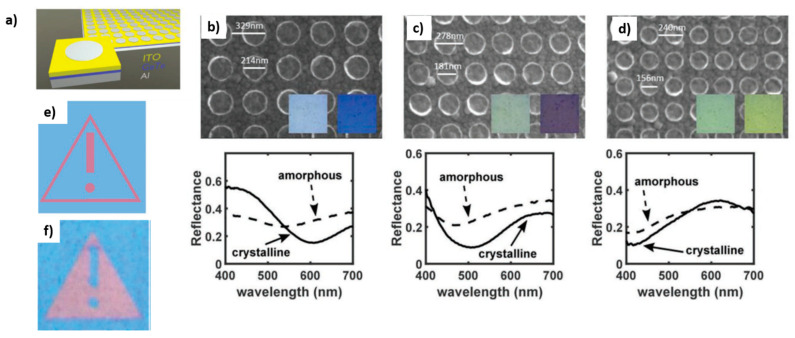
(**a**) Scheme of the structure proposed by Carillo et al. [41] for the obtainment of a colored display, employing a structured metasurface; (**b**–**d**) SEM images and corresponding Reflectance spectra of cyan (**b**), magenta (**c**) and yellow (**d**) pixels. Color variation is obtained by changing morphological parameter of the metasurface (disk diameter and periodicity), as reported in the SEM images; (**e**) optical picture of fixed bi-color displays, obtained by combining different cyan and magenta pixels in the crystalline state; (**f**) optical picture of dynamic bi-color displays, obtained by combining same cyan pixels, some in the amorphous and some in the crystalline state. Reproduced from Ref. [41] with permission from Wiley.

Carrillo et al., instead, proposed a layered structure made of a top aluminum layer patterned into circles deposited on a stack of ITO, GeTe and Al layers [41] (Figure 6a). Al has been chosen because it supports a plasmonic behavior in the visible range and it is easily manipulable, ITO has been chosen because it provides environmental protection from oxidation of GeTe and because it is transparent, while GeTe has been chosen for its phase change properties. In particular, when GeTe is in its crystalline form, it is characterized by a metallic-like behavior with a negative value of the real part of permittivity, which enables us to support and confine a resonant mode in the ITO layer for a specific spectral band. In this way, light with a particular wavelength (equal to the λ of the resonance) is absorbed by the system. This resonant absorption enables the generation of a particular color, which depends on the properties of the structured metasurface (diameter of the Al circles in the top layer, periodicity of the metasurface) and thickness of the ITO layer. By varying these parameters, it is possible to obtain displays characterized by a different color: cyan (strong resonant absorption in red waveband), magenta (strong resonant absorption in green waveband) or yellow (strong resonant absorption in blue waveband) (Figure 6b–d). On the contrary, when GeTe is in the amorphous state, no resonances are supported, no light absorption at a particular wavelength occurs and the whole system is characterized by an essentially flat reflectance spectrum, which leads to a white-like color. So, the phase switching of GeTe enables us to obtain an on/off control of resonance. Experimentally, the authors use these structures for the preparation of fixed bi-color displays, by combining different pixels, obtained from structures with a different thickness/circle dimension, all in the crystalline state (Figure 6e); or dynamic bi-color displays, obtained by combining identical pixels, some of which are maintained in the amorphous (white) state, while some others are converted into the crystalline (colored) state (Figure 6f). In this case, switching and writing are obtained by means of laser scanning.

### 3.3. Colored and Dynamic Displays Based on VO_2_

As it can be seen, the incorporation of chalcogenide within thin-film stacks is a strategy that has been widely explored in recent years to obtain dynamic color tunability in response to phase change transitions. However, some concerns related to the use of GST225, AIST and other chalcogenides can arise: they are quite toxic; they are characterized by a high light absorption capability, especially when in the conductive state, which limits their employment only in the form of very thin film (<10 nm), with a consequent enhancement of difficulties during the fabrication procedure; and the phase transition occurs at high temperature, leading to significant power consumption. More recently, researchers have begun to investigate VO_2_ as a viable alternative, also for applications in the field of dynamically tunable colored displays, that can be exploited also for the storage of graphical information. The main limitations are related to the fact that, in this case, the phase transition is volatile, so images and writings can be stored just for a limited period of time and undergo a spontaneous self-erasing process in few seconds, and that the refractive index variation is greatest in the infrared and microwave spectral range, rather than in the visible. This fact has initially led to the development of displays for the visualization of tunable images with encoded infrared data [42,43]. However, at λ < 700 nm the phase transition produces a maximum change of 60% in n and of 20% in k, enabling the production of tunable colored displays based on VO_2_ also in the visible range, with the advantages of low toxicity, limited power consumption and easy fabrication (for VO_2_ film deposition different techniques are available, ranging from sputtering, molecular beam epitaxy to plasma laser deposition). 

#### 3.3.1. Applications in the IR Range

**Figure 7:**
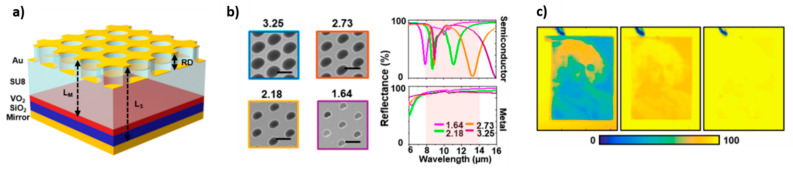
(**a**) Scheme of the structure proposed by Chandra et al. [42] for the encoding of IR images. L_M_ stands for the cavity length when VO_2_ is metallic, L_S_ stands for the cavity length when VO_2_ is in the semiconductor phase and R_D_ stands for the relief depth of the Au-SU8 metasurface; (**b**) SEM images and corresponding Reflectance spectra of different structures with Au holes characterized by different diameters, bot for metallic and semiconductor VO_2_. Scale bar corresponds to 2 μm; (**c**) FTIR scan generated images of the plasmonic surface acquired for (from the left to the right) semiconducting (*T* = 295 K), phase-separated (*T* = 320 K), and metallic (*T* = 360 K) states of VO_2_. Albert Einstein image is 1.3 × 1.7 mm^2^. Reproduced from Ref. [42] with permission of the American Chemical Society.

As regards applications in the IR range, an interesting work is that of Chandra et al. related to a multi-stack structure composed of a complementary gold hole/disk array, a tri-layered cavity spacer (which is composed of a layer of photoresist polymer SU-8, a layer of VO_2_ and a layer of SiO_2_) and a reflective back mirror [42] (Figure 7a).

It is a multilayered cavity-coupled plasmonic system, whose tunability is based on the fact that cavity length changes according to the VO_2_ phase (it increases when VO_2_ is a semiconductor), and, as a consequence, the resonance wavelength of the whole structure is altered. In particular, when VO_2_ is metallic, it behaves like a mirror and the effective cavity length is remarkably shortened, no Fabry-Pérot modes are sustained, and the device reflects about 80% light almost uniformly across the IR band. So, absorption peaks can be observed at low temperatures, but they vanish upon heating. According to the thickness of SiO_2_, VO_2_, and SU-8 layers, as well as periodicity and diameter of the hole/disk array, the position of the Fabry-Pérot assisted localized surface plasmon resonance changes, introducing further tunability. In fact, the design parameters can be optimized to achieve at low-temperature infrared absorption at any desired wavelength. The possibility of using this type of system for the infrared-coded image has been demonstrated by preparing a device with the surface characterized by differently sized holes, which enables us to introduce different resonance wavelengths inside the same structure and different reflectance spectra (Figure 7b). Each hole corresponds to a pixel, and the greyscale values of the desired image can be mapped to hole diameters. Therefore, it is possible to convey information from the visible to the infrared domain. Hyperspectral imaging of this infrared encoded image is then performed: the FTIR spectrum for a single pixel is acquired at room temperature and it is analyzed to obtain the infrared coded image. By heating up the device, the image quality deteriorates until completely disappearing, when the temperature reaches values higher than VO_2_ phase transition, since the reflection of all the pixels simultaneously flattens to 80% (Figure 7c).

An opposite behavior (image appearance in the IR range upon heating, and disappearance upon cooling) is reported by Nagasaki et al., who propose a metasurface composed of cylindrical VO_2_ nanostructures periodically arranged on a sapphire substrate [43]. In this case, the switching between the semiconductor and the metallic phase leads to a modification of the phenomenon at the basis of the observed resonances, with a considerable variation of the reflection spectrum. In the case of the low-temperature phase, the antenna is composed of a dielectric and Mie resonances are excited inside the nanostructure; in the case of the high-temperature phase, the antenna consists of a metal and surface plasmon resonances are excited on the surface of the VO_2_ nanostructures. The main effect obtained upon switching is that at high temperature the reflection peak disappears, and a reflection dip is generated. In this case, by modifying structure parameters, such as cylinder diameter or height, it is possible to obtain different resonance wavelengths. Using this metasurface as a pixel, the authors demonstrate the possibility of obtaining the storage of graphical information by means of a tunable invisible ink. In the final device, they combine pixels obtained from metasurface characterized by disks with different diameters and different reflectance: when *d* = 560 nm pixels have high reflectance, when *d* = 520 nm pixels show high reflectance at low temperatures and low reflectance at high temperatures. The first type of pixels has been used for the background, while the second type for the letters that have to be displayed upon heating. So, the desired message is shown upon heating, while, upon cooling, the image vanishes.

Xu et al. recently reported about another device able to convert a visible image into an IR image through localized optical heating and thermal emission [44]. The device is composed of stacked multilayers made of (from the top to the bottom) 50 nm VO_2_, 30 nm TiO_2_, 850 nm ZnS, 30 nm TiO_2_, 100 nm Au and Si substrate. The top VO_2_ layer confers dynamicity to the whole system, the TiO_2_/ZnS/TiO_2_ trilayer works as a lossless layer, while the bottom Au works as a highly reflective mirror. The absorptance and emissivity spectra of the device have been measured for both the insulating VO_2_ and metallic VO_2_ phases, and, during the insulator-to-metal (IMT) transition, the average emissivity of the device in the 8–14 µm range increases drastically from 0.19 to 0.91, reaching its maximum value ~1 at λ = 10 µm for metallic VO_2_. The VO_2_ phase transition can be triggered by laser illumination, which can be exploited for the recording of thermal patterns, with spatially resolved thermal emissions. The authors demonstrated the writing of both binary patterns (bilevel thermal emission, with a difference of 7 °C) or greyscale patterns (multilevel thermal emission). This multilevel behavior can be obtained by exploiting the hysteresis of VO_2_ phase transition, which means that it occurs at different temperatures during heating and cooling. Due to this hysteresis, two phases and two emissivity levels are allowed at a particular temperature (inside the hysteresis regions, between 53 and 80 °C), according to the fact that it is reached upon heating or cooling. Interestingly, the authors observed that the precise value of emissivity can be controlled by changing the peak temperature during the heating process. During the recording of greyscale patterns, each pixel of the device is written by laser pulses with different durations which enable to obtain different peak temperatures during heating and different emissivity levels.

#### 3.3.2. Applications in the Visible Range

**Figure 8:**
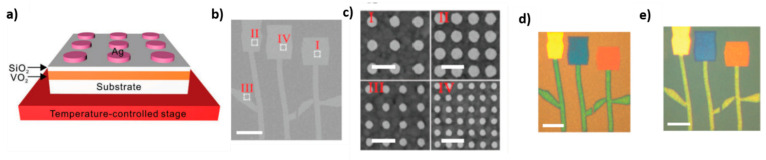
(**a**) Scheme of the structure proposed by Shu et al.; (**b**) SEM image of the pattern prepared for the visualization of tulips image by combining five different structures. Bar = 40 μm; (**c**) SEM images of the four arrays contained in structures I-IV reported in (**b**). Scale bars correspond to 400 nm; (**d**) reflection image of tulips pattern, recorded at 20 °C. Scale bar corresponds to 40 μm; (**e**) reflection image of tulips pattern, recorded at 80 °C. The scale bar corresponds to 40 μm. Reproduced from Ref. [45] with permission from Wiley.

In regard to applications in the visible range, Shu et al. demonstrated the possibility of dynamic color generation in a VO_2_ based device, by integrating plasmonic nanostructures (periodic array of Ag nanodisks) with VO_2_ thin film [45]. A silicon dioxide film is used to separate the VO_2_ film from the silver-nanodisk array in order to reduce the electromagnetic loss and glass is used as substrate (Figure 8a). By tuning the spatial periodicity of the arrays and diameter of the silver nanodisks and thickness of the SiO_2_ layer, various colors can be achieved across the entire visible spectrum. In addition, by exploiting insulator–metal transition of vanadium dioxide, the colors can be actively tuned by varying the temperature. For example, when the disk diameter is 120 nm and the spatial periodicity is 300 nm, the device is greenish at room temperature, and yellow for temperatures higher than 80 °C. Alternatively, when the disk diameter is 100 nm and spatial periodicity is 200 nm, the device is deep green at room temperature, and blue for temperatures higher than 80 °C. In general, when the temperature is raised to 80 °C, the peak shifts to shorter wavelengths and the peak width increases, which leads to the color change. In this work, the authors demonstrated the possibility of constructing temperature-dependent multicolor image (i.e., tulips image) also in the visible range, by combining structures characterized by different geometrical parameters (different nanodisk diameter and periodicity). By increasing temperature to 80 °C, the colors of the image change simultaneously due to the insulator–metal transition of VO_2_, and new colors are generated. Thereafter, when the sample is cooled down to room temperature, the sample resumes its original colors, thanks to the reversibility of the phase transition (Figure 8b–e).

Another proposed structure is the one recently reported by He et al.: a 5 nm thick silver periodic porous layer on the top, a 50 nm thick VO_2_ layer in the middle and a 200 nm thick silver layer at the bottom [46].

**Figure 9:**
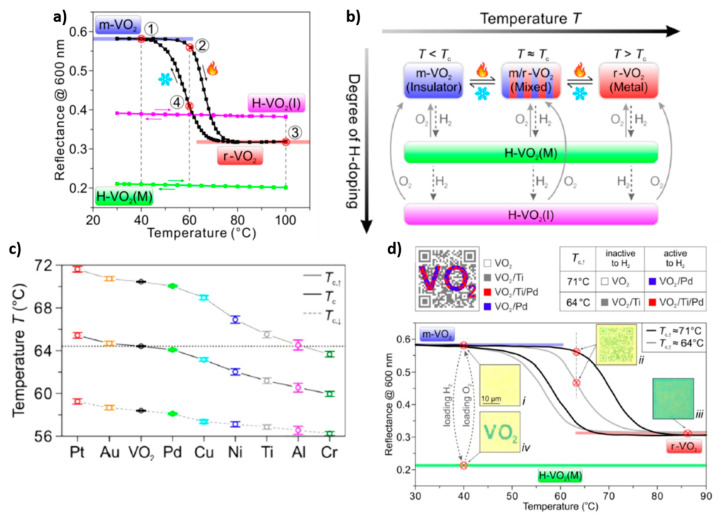
(**a**) Reflectance of the structure proposed by Duan et al. measured at 600 nm and at different temperatures and H-doping levels. H-doping introduces two hydrogenated phases, H-VO_2_(M) (green curve) and H-VO_2_(I) (pink curve); (**b**) possible paths of phase transition of VO_2_ by modifying temperature and H-doping; (**c**) variation of the transition temperature of VO_2_ upon electron-doping. The gray-dotted line indicates the transition temperature of VO_2_ without any surface metal; (**d**) example of dual-key information encryption. Variations of reflectance at 600 nm corresponding to the four states (i, ii, iii, and iv) of the optical double-encrypted display. The two decryption keys are temperature and hydrogenation, respectively. Reproduced from Ref. [47] with permission from the American Chemical Society.

Duan et al. reported the possibility of producing a reconfigurable multistate optical system, by controlling the phase-transition characteristics of VO_2_ with simultaneous stimuli: temperature, hydrogen doping and electron doping [47]. In fact, limited hydrogenation of VO_2_ can result in a metallic phase at room temperature, H–VO_2_(M), while heavy hydrogenation can result in a different insulating phase, H-VO_2_(I), characterized by different reflectance and resistivity, compared to common insulating monoclinic VO_2_. H–VO_2_(M) is characterized by lower reflectance (in the visible range, at 600 nm) and resistance than all the other types of VO_2_ (monoclinic, rutile or H–VO_2_(I)); while H–VO_2_(I) is characterized by a reflectance that is intermediate to those of monoclinic and rutile VO_2_ and higher resistance (Figure 9a). Interestingly, also rutile VO_2_ can be converted into H–VO_2_(M), at a slightly higher temperature in comparison to monoclinic VO_2_, upon moderate hydrogenation, and into H–VO_2_(I), upon further exposure to H_2_. Phase dehydrogenation can also be obtained by exposure to O_2_, converting H–VO_2_(I) into monoclinic or rutile VO_2_, according to the operation temperature and bypassing the H–VO_2_(M) phase (Figure 9b). The authors demonstrate a quadruple-state dynamic plasmonic display by exploiting the VO_2_ tunability in response to a combination of temperature and H-doping. The proposed device is made of stacked Al/Al_2_O_3_ nanodisks, which reside on a substrate composed of Pd nanodots distributed on a 60 nm film of VO_2_ deposited on an Au (100 nm)/Si substrate. The Pd nanodots serve to catalyze the hydrogenation of VO_2_ at low (20–100 °C) temperatures. The height of Al and Al_2_O_3_ disks is fixed at 30 nm, while their diameter and their periodicity can be modified in order to develop structures with different colors. For each structure, 4 different VO_2_ states, with 4 different colors, can be obtained. For example, when monoclinic VO_2_ is blue, H-VO_2_(I) is pink, rutile VO_2_ is red and H-VO_2_ (M) is green. This type of structure can be used to prepare a dynamic color display (as an example, reproducing Vincent van Gogh’s The Starry Night) which can be switched at the four different states, showing vivid color tuning. By depositing thin layers of metals on the surface of VO_2_, it is possible to further control its transition temperature by means of electron doping: when metal with a lower work function (and higher Fermi level) in comparison to VO_2_ is used (for example, Cr, Al, Ti, Ni and Cu), it can donate electrons to VO_2_, facilitating its transition to the metallic phase; on the contrary, when metal with a higher work function (and lower Fermi level) in comparison to VO_2_ is used (for example, Pt and Au), it can accept electrons from VO_2_, preventing its transition to the metallic phase. In the first case, the monoclinic to rutile transition occurs at a lower temperature, in the second case, it occurs at a higher temperature (Figure 9c). The authors demonstrate the possibility of producing dynamic displays encrypted with two-level optical information, with two merged patterns, consisting of a QR code and writing. The display is obtained by combining four different structures: the background is made of structures containing only VO_2_ thin film, the QR code is obtained with VO_2_/Ti systems, while the writing is constructed by VO_2_/Pd nanodots structures (when the writing is overlapped to the background) or VO_2_/Ti/Pd nanodots structures (when the writing is overlapped to the QR code). At the initial state, all the device is made of monoclinic VO_2_ and the display shows a yellowish color with blank information. To read out the QR code pattern, the temperature is utilized as the first decryption key. With increasing temperature, the areas covered by Ti start to become greenish in the temperature range of 64 ± 3 °C, indicating that they underwent a phase transition. With a further temperature increase above 80 °C, all the device cells are converted into the rutile phase, so the QR code disappears and the display shows a uniform greenish color, with blank information. To read out the writing, hydrogen is employed as a second decryption key. Upon loading H_2_, the areas with Pd coverage are converted into H–VO_2_(M) phase and the writing becomes clearly visible (Figure 9c).

**Figure 10:**
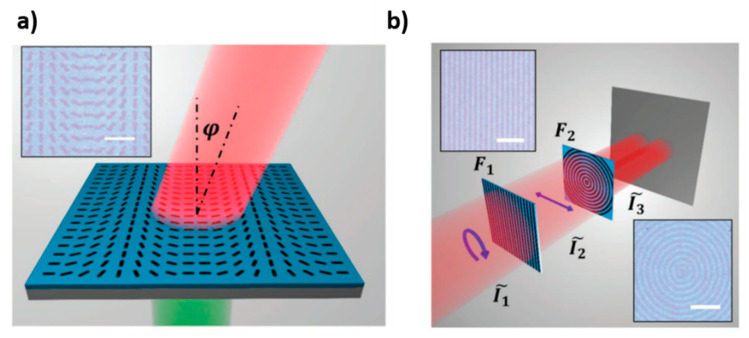
Examples of the employment of VO_2_ based metacanvas used for the writing and storage of optical operators and interaction with light. (**a**) beam steering; (**b**) combination of two VO_2_ based metacanvas, the first programmed and used as a linear polarizer, the second programmed and used as concentric ring grating. Reproduced from Ref. [48] with permission from Wiley.

Dong et al. reported the possibility of using a VO_2_ thin film (~200 nm) deposited on a Si substrate as rewritable metacanvas not only for displaying graphical images, but also for writing photonic operators, in order to use the device for light manipulation [48]. For the storage of graphical information, initially, the device is heated up to a temperature near to the phase transition (all VO_2_ is still in the insulating phase), a laser (λ = 532 nm, 1–11.5 mW, *d* = 1–3 μm) is focused onto the film to locally heat VO_2_ to the metallic phase and storage can occur. It is maintained until the temperature reaches a value low enough to enable it to return to the insulating phase (the VO_2_ phase transition is characterized by hysteresis, so there are different transition temperatures upon heating and cooling, which guarantee a sort of non-volatility) and erasing occurs. The device functionality has been demonstrated for displaying images and, more interestingly, for the writing and storage of photonic operators (Figure 10). In this way, it is possible to obtain a device that is able to manipulate light, for example, beam steering or hologram generation. More complex light manipulation processes can be obtained by combining different devices, storing different photonic operators. For example, the first metacanvas can be programmed as a linear polarizer, which can transform circularly polarized light into linear polarized light, and the second metacanvas can be programmed as a concentric ring grating, which can further transform a light beam into a two-lobe pattern (Figure 10b).

Other stacked non-structured multilayers, among which one is made of VO_2_, can generate different and variable colors, as reported by Wilson et al. [24].

## 4. Optical Switching

PCMs offer a unique set of properties to realize active, tunable and reconfigurable photonic devices. Some of these materials, such as the chalcogenides (GST), owing to their non-volatile phase transition, are suitable for reconfigurable photonic devices in which post-fabrication tunability is desired. Other materials with a volatile phase transition are more indicated for low-intensity optical modulation and dynamic tuning. In particular, VO_2_ is attracting attention for low-power tunable devices because it exhibits an abrupt and reversible change of its complex refractive index at a relatively low temperature of 68 °C. The opportunities for PCM-based optical switches and modulators are endless although many challenges lie ahead. Future research will most likely require a multi-disciplinary effort: material science, chemistry and nanotechnologies will offer solutions to tackle the complex problems of integrating PCMs within commercially-available photonic platforms (e.g., silicon photonics), improving the quality of PCMs with proper alloying and doping, and efficiently introduce the stimuli needed to switch PCMs; fundamental research on hysteresis effects and the dynamics of the phase transition will be key to understand the ultimate limits of PCM-based devices in terms of modulation speed as well as the peculiar behavior of nanostructured PCMs (nanoparticles or nanocrystals); research efforts to develop accurate and fast multiphysics models, which include simultaneously thermal and optical effects, will be key to engineer ultra-compact devices for both photonic-integrated circuits and free-space operation with high modulation depth, low losses, low-intensity stimuli and reduced fabrication complexity.

Various strategies have been so far proposed in order to attain tunability, modulation and switching with photonic devices including PCMs. The operational wavelength in which PCM-enabled tunability has been demonstrated covers the entire spectrum, ranging from visible to THz frequencies. For the sake of clarity, we have classified the design strategies of the literature in three groups: (I) integration of PCMs with silicon-photonics devices, such as waveguides and resonators; (II) planar structures made of multilayer thin films of PCMs combined with films of metals and other dielectrics; (III) photonic nanostructures, such as nanoantennas, plasmonic and dielectric metasurfaces, as well as resonant gratings.

### 4.1. Integration of PCMs within Waveguides and Integrated Resonators

**Figure 11:**
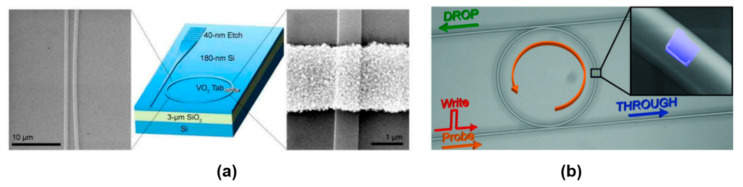
Integrated PCM-based photonic switches. (**a**) Reproduced from [49] with permission from OSA. (**b**) Reproduced from [50] with permission from Wiley.

Optical modulation in silicon devices, such as waveguides and resonators, is a long-standing goal of the photonics research community. The ultimate objective is to realize all-optical signal processing for fast interchip and intrachip communications. So far, the best performances in terms of modulation efficiency have been obtained by embedding plasmonic resonators, polymers, two-dimensional materials, germanium and, more recently, PCMs in silicon-based devices. The typical strategy to achieve tunability and switching in integrated photonics with PCMs is to introduce the PCM in the proximity of a resonator, as illustrated in Figure 11. 

An optical modulator was demonstrated by Briggs et al., in which a 2-micrometer long section of a silicon ring resonator was partially covered with VO_2_ by pulsed-laser deposition, as depicted in Figure 11a [49]. In this work, direct substrate heating was applied as an external stimulus in order to induce the insulator to metal transition in VO_2_ and to trigger the absorption modulation at wavelengths tuned near the resonances of the ring (~1550 nm). This modulation strategy was exploited by Ryckman et al. in a silicon ring resonator partially covered with a PCM patch deposited by electron-beam vaporization of VO_2_ powder [51]. A 10-dB modulation of a cw signal at 1550 nm was activated by a pump laser at 532 nm, which was able to shift the ring cavity resonance by 1.26 nm. Similar modulation efficiency was observed by Rude et al. in a non-volatile optical switch for telecom wavelengths (1550 nm) realized with a silicon ring resonator with an RF-sputtered over-cladding layer of GST225 [52]. Here the phase transition was driven by a pulsed laser diode at 975 nm and the observed switching time for crystallization and amorphization of GST225 was on the order of a few micro-seconds. All-optical switching at 1.55 micron, driven by 25 ns pulses at 1064 nm, was demonstrated by Ryckman et al. in 2013 [53]. The switching mechanism was absorption modulation, with an efficiency of 4 dB/micron, in a non-resonant configuration with a VO_2_ patch covering a section of an Si ridge waveguide, and intracavity phase modulation ~π/5 rad/micron. An electrically driven absorption modulator was proposed in 2015 by Joushaghani et al., in which a VO_2_ film covered a micrometer-long section of a silicon waveguide and it was electrically excited with gold contacts [54]. A modulation efficiency with an extinction ratio of 12 dB was demonstrated with electro-optic bandwidth of ~1.3 MHz, corresponding to a switching time of less than a microsecond. A similar electro-absorption modulation effect was demonstrated by Markov et al. with a significant improvement of the switching time, which was brought down to a few nanoseconds [55]. A non-volatile optical switch, reported in Figure 11b was presented by Stegmaier et al., by depositing a GST225 micro-sized patch on top of a ring cavity [50]. In this configuration, a switching time of ~200 ps with a 1 ps laser pulsed source was demonstrated without optical power bias to keep the device stable at the two states. The on-off switching contrast was of ~ 5 dB with an insertion loss of ~5 dB. A highly-compact, thermally-activated switch based on VO_2_ embedded in a 500-nm long section of a silicon waveguide was presented by Miller et al., with a modulation depth of ~10 dB [56]. According to Zhang et al., a broadband nonvolatile switching at telecom wavelengths can be obtained with an optimized design that exploits GST225, reaching modulation efficiencies of 15 dB and insertion losses as low as 0.1 dB [57].

### 4.2. Integration of PCMs within Planar Multilayer Films

**Figure 12:**
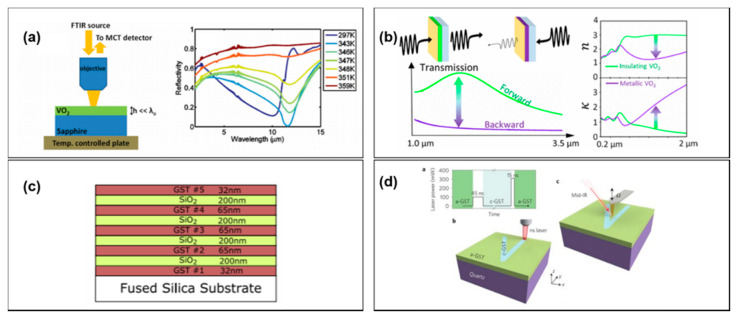
Integration of PCMs within planar multilayer films. (**a**) Reproduced from [58] with permission from AIP. (**b**) Reproduced from [59] with permission from the American Chemical Society. (**c**) Reproduced from [60] with permission from IEEE. (**d**) Reproduced from [61] with permission from Nature Springer.

Optical thin-film filters have a rich history, which dates back to Newton’s measurements of film thicknesses (Newton’s rings), the theory of light as a wave and the first experiments on interference by Young. Although design strategies and fabrication technologies for interference films are quite mature, the ability to endow the optical response of these planar structures with tunability remains an open goal. GST225 and VO_2_ have both been extensively investigated as possible game-changers to reach this goal. Figure 12 reports a literature selection of some of the switching and modulator concepts based on thin planar films.

Early works on optical switching abilities of VO_2_ thin films date back to the late 90s. Beteille and Livage measured thermal-induced switching in vanadium dioxide thin films deposited from vanadium alkoxides [57]. In this work, it was also shown the typical hysteresis of the transition, as well as the possibility to control the transition temperature by properly doping VO_2_ during deposition. Mid- and far-infrared switching was then optimized in films of VO_2_ sputtered on amorphous silica substrates in [57,62]. The optimization was performed by varying sputtering parameters, such as film thickness, gas ratio and substrate temperature. The best performances were observed with film thicknesses of about 120 nm. Transmittance modulation of 75% between the insulator and the metallic state of VO_2_ was measured at 3.5 micron, while reflectance and emissivity variations of ~50% were observed between 8 and 12 micron.

Similar planar structures, based on thin films of VO_2_ and VO_2_O_5_, were also investigated as dynamic optical limiters in the infrared [63]. In these experiments, the IMT transition was induced by a cw infrared laser irradiating the surface of the samples with an irradiance of ~250 W/cm^2^. The limiter performance, with a transmittance decrease from 47% to 28% in the VO_2_ sample, was mainly restricted by the slow response time (~100 ms).

The thermochromic response of VO_2_ is quite sensitive to the deposition process and to the choice of substrate. In [64], substrates of aluminum, silicon and quartz were compared in the sputtering deposition of VO_2_ for tunable emissivity in space applications. It was found that VO_2_ films deposited on an Al display an emissivity dependence on temperature that is opposite to that of VO_2_ deposited on transparent substrates (quartz and Si). In [65] optimized tunability of thermal emission was designed in thin-film multilayer structures made by alternated layers of VO_2_ and metal for applications in smart windows and infrared signature reduction. A very promising application was demonstrated in [58] by Kats et al., in which perfect absorption by critical coupling was achieved in the proximity of the IMT in an ultra-thin film of VO_2_ deposited on top of a sapphire substrate (the structure and the temperature-dependent reflectivity spectra are reported in Figure 12a). This work has shown that a VO_2_ ultra-thin film near the IMT behaves like a metamaterial mixture in which the insulator and metallic phases coexist. The mixture optical response can be easily regulated by applying an external stimulus (thermal, electrical or optical). A large tuning range for reflectance, from 80% to 0.25%, was measured, thus paving the way for applications in bolometers, modulators and controlled thermal emission. Recent work by Wan et al. [59] has demonstrated an optical diode for near-infrared light with broadband asymmetric transmission triggered at high input intensities. The device has been realized with a VO_2_ thin film sputtered on top of a sapphire substrate and covered with a metallic semi-transparent layer (Figure 12b).

Optical switching has been also investigated in GST films (see [12,66] for a recent review). GST225 can be deposited on virtually any substrate or film at low temperatures, and it therefore can be integrated in multilayer structures [60]. The phase transition from amorphous to crystalline induces a significant change of the optical properties, and this modulation occurs on fast timescales, on the order of pico- to nanoseconds. Unlike VO_2_, GST phase change is non-volatile. Crystallization is relatively easy to trigger, while the opposite process, amorphization, requires heating to a significantly higher temperature and it must be followed by a fast quenching.

In [60], a reflective optical limiter was experimentally demonstrated with a multilayer hosting GST films alternated with SiO_2_ films in a photonic-band-edge filter configuration, as schematized in Figure 12c. Recently, an all-optical switch based on layered epitaxial trigonal GST124 was demonstrated for telecom wavelengths with film thicknesses on the order of 100 nm [67]. Reversible optical switching of highly confined phonon–polaritons in quartz was obtained with an ultrathin (~7 nm) planar film of GST326 [61]. The film was activated with laser pulses (see Figure 12d). This demonstration has indicated a novel practical way to realize all-dielectric, rewritable phonon-polariton resonators. 

### 4.3. Integration of PCMs within Photonic Nanostructures 

Photonic nanostructures may display a variety of resonances, whose properties, such as quality factor and modal volume, depending on the shape and size of the structure, on the optical properties of the materials inside the resonator and on the optical properties of the materials surrounding the resonator. For example, in a very simple, but the representative scenario, in which the nanostructure is made by an isolated or an ensemble of very small spherical nanoparticles, the optical properties of the nanostructure, such as near-field enhancement, scattering and absorption spectra, will be ruled by the polarizability of the single nanoparticle. In the dipole or quasistatic approximation, the polarizability can be written as $\alpha =3\epsilon_{\mathrm{bg}}V\frac{\epsilon_{\mathrm{np}}-\epsilon_{\mathrm{bg}}}{\epsilon_{\mathrm{np}}+2\epsilon_{\mathrm{bg}}}$, where V is the volume occupied by the nanoparticle, $\epsilon_{\mathrm{bg}}$ is the permittivity of the background material and $\epsilon_{\mathrm{np}}$ the permittivity of the nanoparticle. Any permittivity change either in the nanoparticle ($\epsilon_{\mathrm{np}}$) or in the environment ($\epsilon_{\mathrm{bg}}$) will induce a change in the optical properties of the nanostructure. The relative change will be larger especially when photonic resonances are excited. This can be realized by introducing PCMs either in the volume of the resonator or in the environment surrounding the resonator. This concept can be applied even to the simplest manifestation of nanophotonic resonance, i.e., the Fröhlich resonance of a very small metallic nanoparticle, which occurs when $\epsilon_{\mathrm{np}}=\;-2\epsilon_{\mathrm{bg}}$. In 2005, Maaza et al. demonstrated reversible tunability of the surface-plasmon polariton resonance of Au nanoparticles embedded in a VO_2_ matrix [68]. The mixture was able to thermally switch, showing a resonance shift from 645 to 598 nm. Similar blueshift effects were observed in mixtures of silver nanoparticles embedded in VO_2_. [69]. In [70], circular and elliptical gold nanoparticles were lithographically fabricated and then covered with crystalline grains of a VO_2_ film. Albeit more complex, the lithographic approach has the advantage to avoid the resonance broadening associated with the dispersion of the particle size that one typically has in self-assembled mixtures of nanoparticles. The thermally induced blueshift of the surface plasmon resonance was measured to be on the order of 80 nm for circular nanoparticles and 200 nm for elliptical nanoparticles. Plasmonic resonance can be also supported by VO_2_ nanoparticles, when the VO_2_ is in its metallic phase. In [71], VO_2_ nanoparticles were obtained by ion-implantation and self-assembly in a silica matrix. The onset of the nanoparticle plasmonic resonance at telecom wavelengths was photoinduced using a pulsed laser at 800 nm (Ti: Sapphire) with pulses having a time duration of ~50 fs and energy of 1–3 μJ. In this work, the dynamics of the phase transition were measured with a pump-probe setup and it was found that the IMT transition occurs on a time scale of about 150 fs. This result paved the way for the development of ultrafast photonic devices based on PCMs. VO_2_ nanoparticles have been extensively studied for energy-efficient thermochromic fenestration, in which solar energy is transmitted below a comfortable temperature and otherwise rejected in order to reduce the use of space heating and cooling [72]. In comparison with VO_2_ films, VO_2_ nanoparticles have been proved to show larger luminous transmittance and deeper modulation of solar energy. The only limitation of VO_2_ for this kind of application is the relatively high temperature for transition, which can be drastically reduced to a comfortable temperature by replacing some vanadium with tungsten [73] or by adding magnesium dopant [74]. The amorphous to crystalline change of GST has been also explored to dynamically control plasmonic resonances [75].

**Figure 13:**
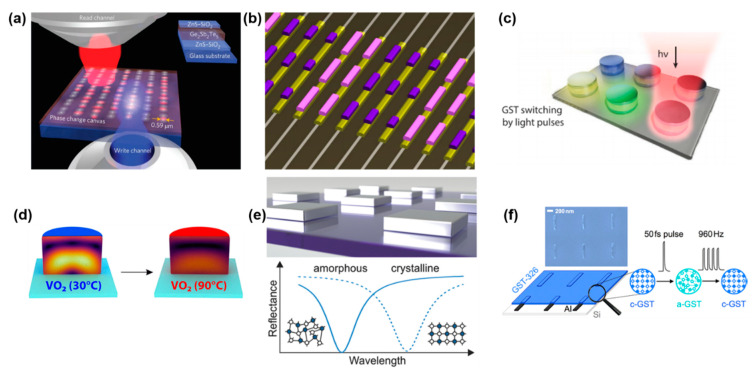
Integration of PCMs within photonic nanostructures. (**a**) Reconfigurable planar thin-film metasurface. Reproduced from [76] with permission from Nature Springer. (**b**) Reconfigurable metasurface based on GST rods. Reproduced from [77] with permission from Wiley. (**c**) Reconfigurable metasurface based on Ge-GST meta-atoms. Reproduced from [78] with permission from Wiley. (**d**) VO_2_-based nanoantennas for metasurfaces in the visible. Reproduced from [79] with permission from the American Chemical Society. (**e**) Tunable perfect absorber based on metal-GST-metal resonances. Reproduced from [80] with permission from Wiley. (**f**) Plasmonic antennas switched with a PCM. Repoduced from [81] with permission from the American Chemical Society.

Besides very small nanoparticles, a plethora of resonant configurations have been investigated, including plasmonic antennas operating in the near and mid-infrared, metallic antennas in far-infrared and in the THz range, dielectric Mie-type resonators, plasmonic and dielectric metasurfaces, metamaterials, as well as resonant gratings. Figure 13 reports a literature selection of representative concepts for PCM-based switches and modulators that have been realized with photonic nanostructures.

The pioneering work of Sun et al. in 2006 [82] has been probably the first demonstration of tunable response in resonant, periodic nanostructures based on PCMs. In that work, modulation of extraordinary optical transmission was experimentally observed in structures obtained with periodic hole arrays on a metal-VO_2_ double-layer thin film structure. Thermal tuning of metamaterial/metasurface resonances were observed at THz frequencies by Driscoll et al. in 2009 inter-twining metallic split-ring resonators with vanadium dioxide [83]. A similar structure was investigated in [84], but in this case, the switching was induced by a combination of thermal and electrical stimuli near the resonance frequency of the split ring resonator (approximately at 1.5 THz), and the measured resonance red-shift was about 20%. By exploiting the hysteretic nature of the VO_2_ phase transition, it was possible to demonstrate persistent frequency tuning of the array using a transient electrical stimulus. Extension of this concept to near-infrared wavelengths was demonstrated by Dicken et al. in 2009 [85]. 

Electro-optical switching of a Fano-type resonance of a plasmonic metasurface was observed experimentally by Samson et al. [86]. In this work, a PCM film of gallium lanthanum sulphide (GLS) was sputtered on top of a resonant plasmonic nanostructure made of asymmetrically-split ring resonators. The electrically-driven transition in GLS provided about a 150 nm shift in the near-infrared resonance, with a transmission modulation contrast ratio of 4:1. This design was then improved by exploiting the more common GST225 PCM in a similar configuration, in which a GST225 film, sandwiched between ZnS/SiO_2_ cap and buffer ultrathin layers, covered the surface of a gold metasurface [87]. In this structure, switching the infrared resonance of the metasurface was obtained by optically stimulating the GST225 phase transition with laser pulses having a time duration of 50 ns and peak intensities of about 0.25 mW/μm^2^. The reversible nature of the switch was also tested with the repeated optical operation, revealing an endurance of 50 cycles before degradation. Infrared tunable resonances were demonstrated in a plasmonic crystal made of gold nanoantennas fabricated on top of a GST225 thin film [88]. The peculiarity of this structure was the sharp nature of the switched resonances, which was associated with a lattice mode of the plasmonic crystal rather than to a localized surface plasmon resonance. A remarkable non-volatile shift of 500 nm was measured. 

Modulation in the THz was proven in 2010 by Liu et al. using a metallic split-ring resonator on top of a VO_2_ film [89]. The modulation efficiency was associated with strong field enhancement provided by the resonators in the gap regions, which become shorted when the VO_2_ switched to the metallic phase. An interesting pump-probe experiment on this structure, in which both the pump and the probe were tuned at TH frequencies, revealed the dynamics of the transmission modulation, which showed a characteristic switching time on the picosecond scale. Proof of principle for tunability of aluminum nanorod antennas was provided in the mid-infrared (~5 μm) using both GST and InSb [90]. The resonance shifting was measured to be 19.3% with a figure of merit, i.e., resonance shift divided by resonance bandwidth, of the order of one. In this work, different configurations were considered, with the PCM on top of the antennas, in the same plane of the antennas and on the bottom. The PCM was selected for its low absorption losses at the resonance frequency of the antenna. In this work, the crystallization of GST and InSb was achieved via annealing on a hot plate. Optical switching was demonstrated by the same authors and in the same type of structure, i.e., aluminum nanorods surrounded by GST326 (see Figure 13f), in [81] by using fs laser source at 800 nm to induce the phase change. Thermal tuning in the mid-infrared was also demonstrated using VO_2_ as PCM in close proximity of a resonant plasmonic antenna array [91]. The Y-shape antennas were fabricated on top of a VO_2_ film of 180 nm using sapphire as substrate, and the volatile modulation was induced thermally. The measured relative shift was about 10% at a wavelength of ~10 μm. 

Complete control over the radiation pattern of nanoantennas can be achieved by judiciously leveraging the refractive index changes of PCMS. In [92] a metasurface of patch antennas was proposed, in which a GST layer is sandwiched between two metallic patches. Exploiting the Kerker condition, in which induced magnetic and electric dipoles interfere either destructively or constructively in the forward/backward directions, and under the application of a stimulus that changes the phase of GST from amorphous to crystalline, the radiation pattern switches dramatically from unidirectional to dipole-like. An alternative way to tune the radiation properties of PCM-based optical antennas in the near-infrared was indicated by Savalyia et al. [93]. The idea is to replace a portion of the volume of plasmonic antennas with a PCM (in the proposed configuration, VO_2_) in stark contrast with the most common approach in which VO_2_ is in proximity, but in the outside body of the nanoantenna. 

Selective absorption and dynamic control of absorption bandwidth in the mid-infrared is of paramount importance for thermal imaging and for efficient microbolometer in many applications, including astronomy, military and surveillance. A perfect absorber based on a patch-antenna array deposited on top of a grounded film of PCM (GST) was proposed by Tittl et al. [80]. The structure, as well as its principle of operation, are reported in Figure 13e. By exploiting the metal–semiconductor–metal plasmonic magnetic resonances in this system, peaks of absorption larger than 90% and with a selective bandwidth of ~1 μm were redshifted by 0.7 µm while maintaining high absorbance. Moreover, 20 μm by 20 μm pixels of the metasurface tuned at different wavelengths in the mid-IR were fabricated, showing the potential application of this technology for multispectral thermal imaging. A similar concept device was proposed by Wang et al. in a VO_2_-based metamaterial absorber, in which the plasmonic magnetic resonance is supported in a one-dimensional gold grating separated by a gold mirror by a thin VO_2_ film [94]. The perfect absorption for TM-polarized light at 5 μm is completely suppressed when VO_2_ switches to the metal phase. A similar configuration, i.e., a resonant array of plasmonic antennas separated from a metallic mirror by a PCM film, was proposed by Cao et al. to realize broadband, polarization- and angular-independent perfect absorption in the visible range [95]. In their work, they indicated GST as the absorbing material for the large imaginary part of its dielectric constant in the visible, and it was predicted that a modest intensity signal of ~95 nW/μm^2^ would be sufficient to switch the GST at the nanosecond scale. 

A different approach to obtain tunable perfect absorption in the near-infrared was proposed by Zhu et al., where VO_2_ was only present in the gap of bow-tie antennas [96]. The antenna array was separated from a metallic mirror by a dielectric spacer of Al_2_O_3_. The advantage of this configuration is the power and time required to switch the PCM are quite reduced, since the PCM only occupies the tiny volume inside the gap of a nanoantenna, in which the field enhancement is very large. The switch was electrically driven by exploiting the antennas, connected to larger electrodes, as heating elements. The measured modulation depth was 33% with a recovery time on the order of a millisecond. A theoretical study on VO_2_-loaded dipole antennas was presented by Tognazzi et al. [97]. It was shown that the switching capabilities of this type of hybrid antenna are ruled by the antenna’s arm length, and that smaller VO_2_ volumes in the gap yield larger extinction-efficiency contrast as well as larger intensities of the resonant peaks. Furthermore, these structures are weakly sensitive with respect to changes of position and size of the gap, revealing good robustness against small fabrication defects. 

Arrays of metal–PCM–metal resonators were also proposed as low-power and fast mid-infrared beam steering devices using GST as PCM [98]. A steering range of 11 degrees in transmission and 22 degrees in reflection was numerically predicted with a steering response on the nanosecond scale and power density of a few μW/μm^2^. Reversible beam steering was experimentally demonstrated in the near-infrared (1.55 μm) with plasmonic 1D antennas fabricated on top of a GST film [99]. The array reflection switched with a 44% efficiency from anomalous to specular, in response to the phase switch induced on the GST film. 

PCMs are also able to control the polarization state of electromagnetic waves. A switchable THz quarte-wave plate, thermally driven, was presented by Wang et al. [100]. The structure was a metasurface obtained with a periodic array of asymmetric cross-shaped resonators, with VO_2_ pads fabricated at the terminals of the crosses. The operational bandwidth, in which the metasurface acted as a quarter-wave plate was able to transform linearly polarized light into circularly-polarized light, blue-shifted by ~10% upon the application of a thermal stimulus. 

Proof-of-principle transmissive tunable metasurfaces were presented by Dong et al., using an array of gold square pillars on top of a GST film and an indium–tin–oxide layer [101]. A 10% shift of the antennas’ resonance was experimentally demonstrated by thermally annealing the sample and the possibility to tune electrically was demonstrated to be feasible, since the ITO layer has a negligible effect on the transmission properties of the metasurface. 

A significant breakthrough for reconfigurable and tunable nanophotonics and, more generally, for the dynamic control of free-space propagating optical fields, was introduced by Wang et al. in 2015 [76]. It was proven that a single film of GST, properly stimulated by engineered trains of optical pulses, can be tailored to function as a metasurface with an almost arbitrary optical response and functionality (see the artistic rendition of the concept in Figure 13a). A variety of devices were demonstrated with this technique: visible-range reconfigurable plates, a lens with subwavelength focus, a greyscale hologram and an all-dielectric metamaterial with on-demand reflection and transmission resonances. 

Owing to their large refractive index, PCMs, and GST in particular, lend themselves naturally to the design of Mie-type resonators and all-dielectric metasurfaces based on Mie resonances. An all-dielectric reconfigurable metasurface was demonstrated by Karvounis et al. with visible/near-infrared switching contrast of ~7 dB in reflection and transmission [102]. The metasurface consisted of a high-contrast, subwavelength grating with sharp leaky-mode resonances and the 10% measured resonance shift was laser-induced. An active metasurface concept was proposed by Chu et al. [77]. The idea is to engineer PCM metamolecules (based on GST rods, as reported in Figure 13b) with a tailored optical response and functionality, including the tuning of anomalous reflection in gradient metasurface for beam steering and the tuning of the electromagnetically-induced-transparency resonance. A full-phase control in all-dielectric metasurface was achieved by Leitis et al. using hybrid Ge-GST Mie resonators as building blocks for the active metasurface, as illustrated in Figure 13c [78]. In particular, a tunable infrared Huygens’ metasurface was presented with dynamic, programmable phase control and large transmission in the infrared. The phase control was achieved via ultrashort laser pulses at 660 nm. Reconfigurable metasurfaces with very large switching contrast (~30 dB) in the mid-infrared have been recently presented by Shalaginov et al. [103]. In this work, varifocal lens design has been demonstrated in which a variable focal plane can be obtained by selectively excite different meta-atoms of the meta-surface. The Huygens’ GST meta-atoms have been engineered with various regular shapes, such as ‘I’, ‘H’, and “+” shapes. PCMs have also been used to tune the Mie resonances in semiconductor metasurfaces. For example, recently, a tunable Mie-resonant dielectric metasurfaces was proposed for infrared light, in which a VO_2_ film underneath an array of Mie-resonant silicon nanocylinders was able to switch transmission by two orders of magnitude [104]. It has been also shown that such a metasurface can be tailored to produce spectrally tunable near-perfect absorption. 

PCM-based dielectric metasurfaces have been also investigated for applications involving visible light. Recently, it has been theoretically predicted that modulation of visible light may be possible with VO_2_-based metasurfaces, exploiting a diverse number of design strategies to increase light interaction through hybrid resonances in which both lattice effects and localized resonances conspire to boost the light-matter interaction [105]. An experimental demonstration of tunable Mie-resonances has been recently provided by Kepič et al., with tunable VO_2_ nanoantennas supporting Mie-resonances in the visible and near-infrared range (Figure 13d) [79]. Moreover, plasmonic resonances were also observed when the VO_2_ was switched to its metallic phase. Scattering and extinction modulation depths were measured on the order of a few dBs, and they were induced both thermally, with a temperature-controlled stage, and optically, by using a cw laser. 

## 5. Photonic Memory Devices for Information Storage and Computing

Initial applications of chalcogenide PCMs were focused on data storage, leading to the development of rewritable optical discs (DVD or blue-rays) or non-volatile phase-change electronic memories (phase-change random access memories, PCRAMs). Data storage is achieved thanks to variations of material reflectivity in the first case, and thanks to electrical conductivity changes (the resistance value of a PCM memory cell is typically around 10^3^ ohms for crystalline state and 10^6^ for amorphous state) in the second case. These applications are at an advanced development stage: PCM-based rewritable optical discs are already commercialized and excellent reviews can be found in the literature regarding PCRAMs [106,107,108,109,110]. They are very promising alternatives to more common Flash memories, thanks to their ultrafast operation (on the time scale of nanoseconds, that is orders of magnitude fasters than Flash memories), high reproducibility, high cycling endurance (the possibility of reaching 10^12^ switching cycles by means of electrical pulses has been demonstrated), low power consumption, especially thanks to the non-volatility of the phase transition, and good scalability of the material to the nanoscale. However, PCRAMs are characterized by some drawbacks, such as resistance drift over aging (due to long term instability of the amorphous state) and difficulty of reaching with precision the desired resistance level (often it is necessary to check and verifying if a target value has been reached, following a “program and verify” method, which enhances the whole process complexity). In addition, PCRAMs are still linked to von Neumann architecture inside computers, which separate, in time and space, the operations of processing and memory.

More recently different research groups have begun to study the possibility of using PCMs, in particular GST225, for obtaining photonic memories, in which data are stored thanks to the variation of light transmission along a photonic circuit. In this case, the obtained devices are characterized by non-volatility, high cyclability (10^6^ cycles) and high operation speed, which is further increased by the fact that both information storage and transfer can be performed by optical means. For example, PCRAMs typically require a writing pulse of 50–100 ns and reading pulse of 10 ns, while for PCM photonic memories 10 ns and 500 ps are enough for writing and reading, respectively [111]. PCMs have the advantage of being easily manipulable at the nanoscale and they can guarantee good compatibility with semiconductor processing and on-chip optical interconnects typical of photonic circuits, leading to high-speed information transfer, large bandwidth and low loss residual crosstalk.

**Figure 14:**
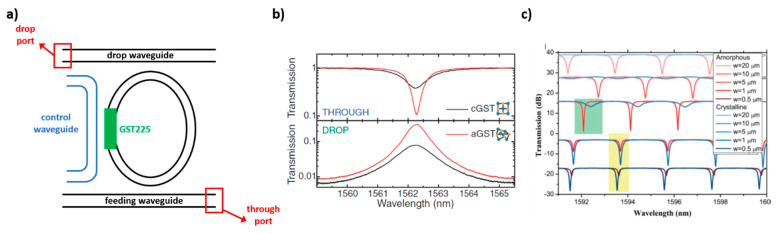
(**a**) Examples of a commonly employed architecture for the preparation of memory cell, composed of a ring resonator containing a switchable GST225 unit, a feeding waveguide and a drop waveguide. In the work of Pernice et al. a control waveguide is used to induce phase transition [112]; (**b**) variation of transmission recorded at the through port (up) and at the drop port (bottom) upon GST225 switching. Laser pulses are used to induce phase transition [50]; (**c**) variation of transmission at the through port upon GST225 switching, according to different width values of the GST225 patch. It is possible to modify the ring resonator parameters (radius and gap with the feeding waveguide) in order to obtain “direct switching”, as highlighted in the green region, and “inverse switching”, as highlighted in yellow regions [113]. Reproduced from Refs. [50,113] with permission from Wiley.

The first proposal of using Ge_2_Sb_2_Te_5_ for obtaining a photonic memory comes from Pernice et al. in 2012 [112]. The proposed architecture is made of a microring resonator coupled to a nanophotonic bus feeding waveguide, a control waveguide and a drop waveguide. The waveguides are made of SiN and on the top of the microring resonator is a patch made of GST225, which introduces dynamicity into the system (Figure 14a). In fact, light that passes through the control waveguide can evanescently couple to the ring resonator, interacting with the GST225 patch. In this way, it causes a temperature enhancement of the GST225 layer and induces a transition phase. For this tuning, high-intensity laser pulses are used, with λ = 700 nm and a duration of 600 fs. The architecture can be optimized in a number of ways: by varying the gap between the control waveguide and the ring resonator it is possible to regulate the degree of the heat transfer and, so, the degree of the phase change, and by suspending this portion of the ring resonator it is possible to improve thermal isolation of the GST225 layer, enabling to reach a temperature high enough to undergo quickly crystallization or amorphization processes. When GST225 is in the crystalline state, it is characterized by high light absorption capability, on the contrary, when it is in the amorphous state, it is characterized by low absorption. When some probe light (of the proper wavelength, in resonance with the ring resonator) flows through the feeding bus waveguide, it evanescently couples to the ring resonator, which absorbs it in a different way, according to the GST225 phase. So, by controlling the GST225 phase it is possible to control the modal profile of the propagating wave in the ring resonator. When GST225 is amorphous, light is largely confined into the SiN portion of the ring waveguide and it propagates without significant loss. On the contrary, when GST225 is crystalline, it becomes more absorptive, and light is confined close to the GST225 portion of the ring waveguide, increasing the optical loss through the ring resonator. As a result, the transmission that can be detected at the readout port of the drop waveguide undergoes significant variations according to the GST225 state. Upon crystallization of the GST225, the transmission at the drop port reaches lower values. Transmission variations can be detected also at the through port, with an opposite behavior after GST225 crystallization, i.e., higher transmission values at the through port for crystalline GST225. By switching between amorphous and crystalline phases, it is possible to vary continuously the light transmission through the ring resonator between 0 to 90%. Therefore, by assessing memory levels to selected levels of transmission, it is possible to obtain multilevel memory device. Writing and storage of information are performed thanks to GST225 crystallization, while erasing is performed by means of amorphization. Reading out, instead, is performed without altering the GST225 state, by means of weak probe laser pulses from the input bus waveguide, and it can be performed both at the through or at the drop port. 

The same architecture, i.e., one ring resonator coupled to 2 parallel bus waveguides, is used also by Stegmaier et al. [50]. In particular, it was demonstrated that by controlling the crystallographic state of the GST225 patch on the ring resonator it is possible to control the flow of the on-resonance light inside the device: when GST225 is the crystalline state light is directed to the through port, while it is directed to the drop port when it is in the amorphous state (Figure 14b). In this case, amorphization is achieved by using 2 pulses at high energy (but low enough to not damage the material), while crystallization is obtained stepwise, using a train of pulses with decreasing energy (from 38 to 19 pJ), which enable a slow cooling of the material and guarantee a sufficient time to achieve an ordered arrangement of GST225 atoms. In addition, by modulating the power of the pulses inside the train used for crystallization, it is possible to access intermediate crystallization levels, leading to gradual variation of transmission and intermediate memory levels.

Similar results are obtained using similar devices, based on different architectures (one [14] or more ring resonators [111] coupled to only one bus waveguide, double-ring resonators in series coupled to three waveguides [114], etc.). The operational principle of all these devices is based on the fact the attenuation coefficient of the GST225 in the crystalline state is higher than that obtained for the amorphous state. In fact, as demonstrated by Rios et al., this behavior is independent of the employed architecture [114]. Here the authors tested three different architectures: a ring resonator (with a GST225 switching portion) coupled to one bus waveguide, a micro Mach–Zehnder interferometer (it is composed of two beam splitters and two mirrors, along one of the two possible paths a GST225 patch is deposed on the top surface of the waveguide) and a balanced splitter (one of extremity contains a GST225 switching portion). For all the three structures, transmission measurements have been performed under three different conditions: (i) before deposition of the GST225 on the surface of Si_3_N_4_ waveguides in order to estimate losses due to the waveguides, (ii) when GST225 is in the amorphous state and (iii) when GST225 is in the crystalline state. The variation of the amount of light transmitted in different conditions is linked to the losses recorded inside the structure, and it is proportional to an attenuation coefficient. The attenuation coefficient obtained in the case of crystalline GST225 is higher than the one obtained for amorphous GST225, confirming its more absorptive behavior. It was also demonstrated that the attenuation coefficient is higher for the wider GST225 patch (linear correlation with GST225 width). Furthermore, in this work, the authors proved that it is possible to build a memory device by exploiting a ring resonator coupled to only one bus waveguide, which works both as input and read-out through a port. Interestingly, the authors noticed that when the attenuation inside the ring resonator changes, also the resonance condition changes. In other words, also the position of the resonance peak can be used to retrieve the GST225 state. In addition, the results of this research indicated that it is possible to engineer the ring resonator (modifying its radius, the width of the GST225 patch and the gap between the ring resonator and the bus waveguide) in a way that it is possible to obtain both transmission decrease (direct switching) or increase (inverse switching) upon GST225 crystallization (Figure 14c). This fact is particularly interesting because, not only does it enable to perform data storage by assessing different memory levels to different transmission levels, but it also allows us to implement logic operations by combining direct and inverse switching arrays inside the same device. 

**Figure 15:**
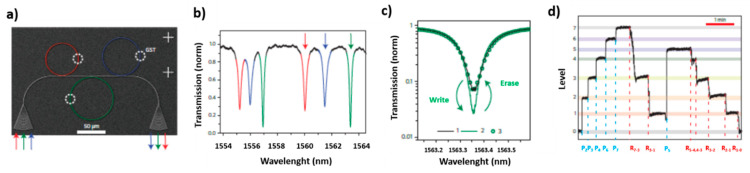
(**a**) SEM image of the architecture of the system used by Rios et al. as a memory device, composed of three differently-sized ring resonators containing a switchable GST225 unit, combined with a feeding waveguide; (**b**) measured transmission at the through port: a group of three distinct resonances, corresponding to the respectively colored rings, are observed; (**c**) variation of transmission at the through port upon GST225 switching, for one individual element. Writing is obtained through amorphization, erasing is obtained through crystallization; (**d**) multilevel operation: 7 different levels can be reached inside the device, by applying laser pulses P_i_ characterized by different power (*E_P*1*_* = 372 ± 12 pJ, *E_P*2*_* = 415 ± 13 pJ, *E_P*3*_* =465 ± 13 pJ, *E_P*4*_* = 524 ± 14 pJ, *E_P*5*_* = 561 ± 14 pJ, *E_P*6*_* = 585 ± 14 pJ and *E_P7_* = 601 ± 15 pJ). Each level is accessible in consecutive (both ascending or descending) or arbitrary order. R_n−__m_ indicates a partial erasing process. Reproduced from Ref. [111] with permission from Nature Springer.

Rios et al. in another work demonstrated the possibility of achieving multilevel and multi-bit data storage, using a device composed of three memory cells (three ring resonators with a GST225 patch) coupled to one bus waveguide [111]. The three-ring resonators are characterized by three different radii, so three different resonance wavelengths. Their different dimensions enable independent access to different cells, both for writing (performed through amorphization, using a high intensity 15 ns laser pulse), erasing (performed through crystallization, using a train of 50 ns pulses of decreasing power) and reading out (performed using lower power 500 ps pulse, which does not modify the GST225 state). Data storage is encoded in the amount of light transmitted along the bus waveguide and read at the through port. When GST225 is in its amorphous state, low values of optical loss are recorded inside the ring resonator (excited at a particular wavelength), and it is possible to record at the through port lower values of transmission in comparison with the same structure with GST225 in the crystalline state. In addition, by properly modulating the energy of the pulses for writing/erasing it is possible to reach intermediate levels characterized by a mixture of crystalline and amorphous regions, which exhibit transmission properties lying between fully-crystalline (level 0) and fully-amorphous (level 1) ones. The authors experimentally demonstrated the possibility of obtaining up to 7 different levels, which can be reached both serially and randomly (Figure 15). However, the number of levels that can be achieved in one device is determined by the difference in transmission between the highest and the lowest level and by the signal-to-noise ratio of the read-out process: it can be increased by using larger memory cells and readout pulse with higher energy.

**Figure 16:**
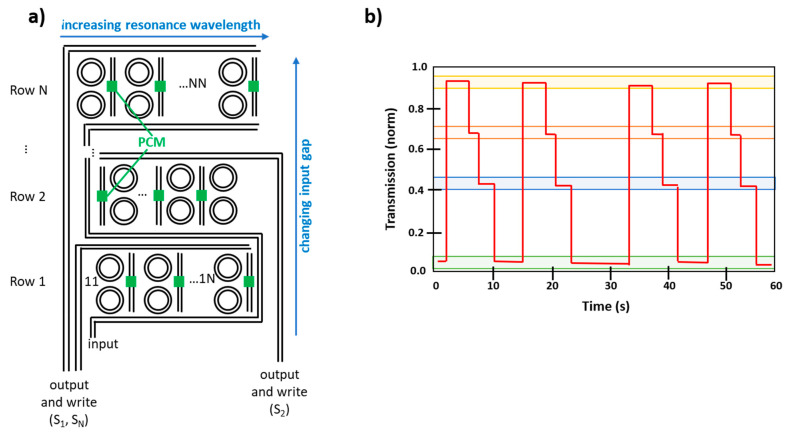
(**a**) Sketch describing the operation principle of the photonic memory proposed by Feldmann et al. [114]. Several PCM-cells are combined into rows and can be addressed individually; (**b**) Multilevel operation of a single memory cell. The phase-change material can be reversibly switched between four clearly distinguishable levels, characterized by different transmissions.

Li et al. proposed an alternative method to obtain multilevel switching, which enables to achieve inside a single cell 34 levels and to prepare memory device of more than 5 bits [115]. The exploited architecture is very simple: A Si_3_N_4_ waveguide on which a patch of GST225 has been deposited, together with an ITO protective layer. As usual, by controlling the GST225 phase, it is possible to control the amount of light transmitted along the waveguide, considering that GST225 is more absorptive when crystalline. The main advantages of this method are that it enables to reach a high number of non-volatile levels, it is very fast (operation last less than 250 s) and a low amount of energy is consumed. It is based on a double-step single optical pulse, which allows us to control the final level of the cell independently of the previous state. In this case, writing is obtained by means of gradual amorphization, while erasing is achieved by means of crystallization. The writing programming pulse is a 20–50 ns rectangular pulse of variable energy, which controls the % of amorphous material and the transmission along the waveguide. Higher power corresponds to higher amorphization and higher transmission. The erasing pulse, instead, is divided into two steps: a first, high amplitude, 50 ns pulse, which serves for heating GST225 above crystallization temperature, followed by longer (200 ns) and lower-amplitude (37% of previous amplitude) pulse, which serves to induce crystallization. Actually, this double-step pulse can be exploited alone to reach different levels inside the single cell, eliminating the first programming pulse, reducing further time operation. In fact, the first high amplitude step is able to erase any previous GST225 state, while the second longer and lower amplitude pulse can control the amount of crystallized fraction, according to its duration. Higher duration corresponds to a higher amount of crystalline phase and lower transmission. The preparation of a device with a furtherly increased memory capacity of 512 bits has been recently demonstrated by Feldmann et al. [114]. The device is a memory in the array format, composed of 16 × 16 cells (16 rows, each containing 16 cells). Each cell is formed by two microring resonators in series coupled to three bus waveguides (made of Si_3_N_4_ on SiO_2_, deposed on silicon carrier wafer): one input waveguide, one perpendicular connection waveguide, and one output waveguide. The input waveguide is the same for the whole device, passing all the cells along all the rows. Each row, instead, has a proper output waveguide, leading to 16 output waveguides in the whole device. The connection waveguides contain a GST225 patch on their top, one for each cell (Figure 16a). Each cell is characterized by a two-bit capacity, since by controlling the phase transition of the GST225 patch, it is possible to achieve 4 levels. The two resonators inside a single cell are characterized by the same radius and therefore the same resonance wavelength, but it is different for all the cells inside one row. In this way, an individual cell can be addressed by selecting light wavelength, both during the programming (changing the GST225 phase) and the reading process. The reading step proceeds as follows: low-intensity light traveling along the input waveguide is evanescently coupled (according to its wavelength resonator radius) to the lower resonator and the connection waveguide of a single cell; after passing the connection waveguide, light is coupled to the output waveguide of the proper row through the upper resonator of the same cell and it is finally read out at the output port of the corresponding line (selecting the appropriate wavelength). According to the crystallographic state of the GST225 patch on the connection waveguide, which is the real memory element of the cell, the amount of light transmitted at the output drop waveguide can be controlled. The programming step is linked to GST225 phase transition and it is obtained by letting high-intensity light flow through the output waveguide (selecting the desired row and wavelength) and coupling it to the upper resonator and the connection waveguide. Depending on the intensity of the laser pulse, four different crystallization levels can be reached (full crystallization for 550 pJ) (Figure 16b). Specifically, crystallization is achieved by means of multistep pulses (similar to the method proposed by Li et al. [115]) composed of one pulse of 50 ns and about 200 pJ, which is responsible for material melting at a temperature higher than crystallization temperature, plus two pulses of lower energy and variable duration (from 0 to 300 ns), which enable to control the degree of crystallization. Higher duration corresponds to a higher crystallization, which leads to lower transmission of light at the output port during the read-out process. The functionality of the proposed device has been successfully demonstrated for the memorization and reproduction of a colored image in 4 colors (2 bits), with high precision. 

**Figure 17:**
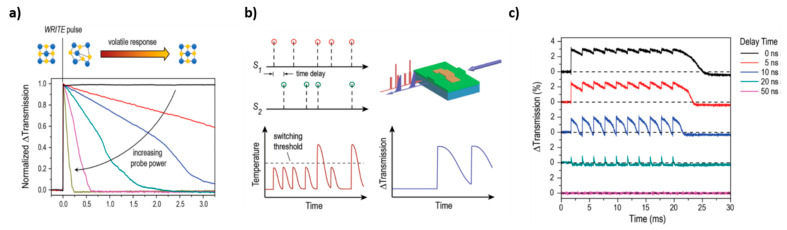
(**a**) Variation of the optical transmission with increasing optical probe power. At low probe powers (black line), the device remains in the amorphous state for nonvolatile operation, while increasing the probe power causes recrystallization of the GST225; (**b**) detection of coincident events between two signals using volatile phase-change memory. Only when two pulses overlap in time, the combined optical power surpass the threshold required to reach an amorphous state (higher transmission level) and can be detected by the device; (**c**) multipulse device dynamics using two pulse trains with varying time delays between them. When the two pulse trains are overlapped in time, the transmission is at a maximum, indicating the two signals are correlated. Reproduced from Ref. [116] with permission from Wiley.

Until now it has been exploited the non-volatility of the GST225 phase transition, to store information in a permanent way inside a non-volatile memory device. In order to induce erasing of the stored information, it is necessary to actively operate on the device, by making light flow along with the device and making occur the GST225 phase transition in the opposite sense. Indeed, in this way, it is possible to mimic only the long-term memory, but not the short-term memory behavior, typical of biological neurons and synapses. Obtaining artificial memory devices that are able to sustain, on-demand, both behaviors will be a further step towards the development of neuromorphic computers. In this contest, Youngblood et al. demonstrated that it is possible to control the duration of retention of phase change inside GST225, by applying a high-energy writing pulse and another high (variable) energy probe pulse [116]. In this case, the probe pulse can also modify the GST225 phase, and it is possible to control the retention time of the previously stored information, by changing its energy. The prosed device is a Si_3_N_4_ waveguide on which 2 μm-long and 10 nm-thick GST225 patches are deposited, covered by a 10 nm ITO protecting layer. The device has been annealed, so at the beginning, the GST225 is in the crystalline phase. By applying a high-intensity writing pulse it is possible to induce gradual amorphization. In order to reach a volatile behavior, the applied probe pulse has to be characterized by energy high enough to perturb GST225 and modify its phase, inducing recrystallization (higher crystallization for higher power). A new value of transmission, which gradually lowers, is obtained at the read-out port, which depends both on the GST225 state after the first writing pulse (i.e., power of the writing pulse) and the power of the probe pulse. In particular, the higher the power of the probe pulse, the faster the recrystallization process, and transmission variation is more pronounced (Figure 17a). The same effect is obtained in the case of low writing power. Thus, by controlling these two parameters, it is possible to optically control data retention time, controlling volatility from years (low probe pulse and high writing pulse) to milliseconds (high probe pulse and moderate writing pulse). This strategy has been exploited to implement inside a photonic device also an example of data computing process: the detection of coincident events between multiple inputs. Coincidence detection is based on the fact that GST225 amorphization can be pursued only when a sufficiently high temperature is obtained. So, when low-energy pulses are used for writing, two pulses must be simultaneously applied (coincidence pulse) (Figure 17b). By monitoring transmission at the read-out port, according to the delay between the two writing laser pulses, it is possible to record some variations: in the case of coincidence (0 ns delay) transmission remains constant at a high value, with small spikes; as delay increases lower values of transmission are obtained (indicating that amorphization has been only partially obtained) and duration and amplitude of transmission spikes decrease. In the case of not correlated pulses, amorphization is not even reached and transmission remains zero (Figure 17c).

The possibility to achieve a non-volatile regime for data storage has been demonstrated by the same authors also inside a device made of a ring resonator (containing a GST225 switching patch) and coupled to two waveguides.

**Figure 18:**
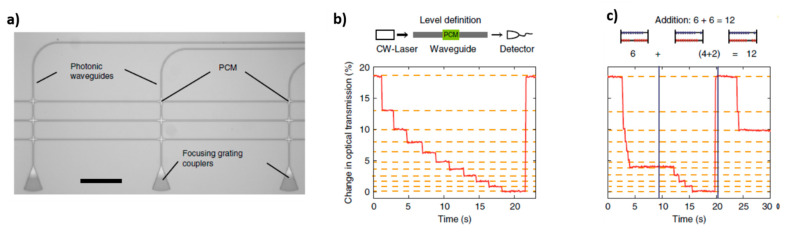
(**a**) Optical micrograph of a crossed-waveguide photonic array used as a memory device for the implementation of arithmetic calculation. Scale bar is 100 μm; (**b**) level definition in a base-ten device; (**c**) example of addition: with the transmission levels defined in b), ‘6 + 6 = 12’ is calculated. By analogy to the operation of an abacus, a carryover is performed when the tenth level is reached, and the cell is reset to its initial state. The result ‘12’ is obtained from one carryover, which can be read in another cell (the corresponding to “tens”), and the final state of the illustrated PCM-cell, which is at level 2. Reproduced from Ref. [117] with permission from Nature Springer.

The capability of simultaneously store and process information by means of a photonic device based on PCMs has been demonstrated also by Feldmann et al., who reported an all-optical abacus-like calculating unit [117]. Abacus provides two of the most basic functions of a computer, processing (calculation) and memory (storage), simultaneously and in a single device, enabling to overcome time limitations of modern computers based on von-Neumann architectures, which separate, in time and space, these operations and require further data transfer and connections. The proposed device is a rectangular waveguide array with a PCM cell at every waveguide crossing point. Each PCM cell with a straight waveguide corresponds to a single processing unit, which can be independently switched thanks to a two-step method (Figure 18a). The pulses of the two steps are launched into the photonic array via two orthogonal waveguides, superimposing at the crossing incorporating the PCM-cell that has to be switched, and they are characterized by energy such that only when they arrive together, the PCM heats up sufficiently to induce a phase transition, similarly to what reported in the case of coincidence detection. In the case of multiple rows and multiple columns array, a simple transmission measurement is no longer appropriate for reading, since the optical transmission along any row or column in the waveguide array is influenced by all the phase-change cells lying on the same row or column. In this case, a two-step pulse scheme is used also for the read-out process: after a PCM-cell has been set to a specific level after the writing process (data processing and memorization), a reset operation consisting of two overlapping pulses is applied. The change in transmission depends on the initial state of the PCM; thus, by sending a reset two-step pulse to the crossing of interest and detecting how much the transmission changes, we can selectively read the state of each PCM-cell in the array in a destructive manner. As PCMs the authors tested both GST225 and AIST, without noticing significant differences. 

In this configuration, each PCM cell represents a single place value, corresponding to the different rows of the abacus, while the abacus beads are represented by ‘quanta’ of crystallization in the PCM (in the form of transmission variation). If we work in base 10, the first cell represents ones, the second tens, the third hundreds, and so on. The number of the base, instead, is linked to the number of crystallization levels that can be reached, and depends on the energy of the laser pulses used until complete crystallization (in the case of base 10, the PCM can be fully crystallized by applying 10 pulses, as shown in Figure 18b). The sliding of a bead to the right (increasing value)/left (decreasing value) is thus represented by stepwise crystallization/amorphization. With this device, the authors demonstrate the possibility to perform all the arithmetic operations: addition, multiplication (in the form of successive addition), subtraction (exploiting numbers complements approach) and division (in the form of repeated subtractions). In Figure 18c, the case of 6 + 6 addition in base ten is reported. Starting from state 0, pulse sequences equivalent to the first summand are sent into the waveguide which sets the first PCM-cell to level six. Then the second summand is added by sending the corresponding (six) pulses. When reaching the tenth level (after four pulses), the first PCM-cell is reset to level 0 (by inducing amorphization) before the rest of the input sequence (two pulses) is applied, by inducing new crystallization. While resetting the cell, one crystallization pulse is sent to a second PCM-cell representing the next highest order multiple of the base to store the carryover information, in this case, “tens” (not shown in the graph). At the end of the calculation, the shown PCM-cell is at level 2 while the second PCM-cell is at level 1, revealing the expected answer of 12. 

A similar device composed of multiple phase-change cells can be used also to perform direct multiplication, by encoding the information related to the first multiplicand into the transmittance level of the cell after the first writing pulse, and the value of the second multiplier into the value of the power used for the “reading” (*P_in_*) pulse. In this way, the power of the pulse of P_in_ at the output port, P_out_, depends on the value of transmittance of the waveguide and represents the result of the multiplication, *P_out_* = *T*((*P_Write_*) × *P_in_*). A similar device composed of multiple phase-change cells can be used also to perform matrix–vector multiplication (using memory cells in series and combining the output signals containing the result of each multiplication, carried out in different cells, by means of beam splitter), which is a basis of many operations of artificial intelligence [118].

Cheng et al. demonstrated that photonic phase-change cells can be used also for the preparation of programmable optical logic devices, based on both “OR” and “NAND” logics [119].

Cheng et al. take a further step forward in the development of photonic neuromorphic computing, demonstrating that photonic synapses obtained by incorporating multiple, small, and discrete GST225 islands on tapered Si_3_N_4_ waveguides can mimic the behavior of biological synapses [120]. The employment of multiple islands instead of a unique patch enables better control of the interaction between the optical pulse and GST225 and better control of its phase transition. In biological brains, a neuron (called pre-neuron) generates action potentials (spikes, fire time *t_pre_*) that propagate along the axon and are transmitted through a junction to the next neuron (called post-neuron) that generates the postsynaptic action potentials (fire time *t_post_*). The junction is the synapse, which is characterized by a synaptic weight (*w*) determining the communication strength between the two neurons. At the basis of memory and learning in the human and animal brain, there is “synaptic plasticity”, that is the capability of changing dynamically synaptic weight, defined as Δ*w* = *f* (*t_post_* − *t_pre_*) = *Ae*^−^^Δt/^^τ^, where A and τ are constants and Δ*t* is the time delay between pre- and post-synaptic signals. In the proposed photonic synapse, the synaptic weight is determined by the level of transmission of the waveguide. In particular, the authors demonstrated that the proposed photonic synapses are effectively able to modify the synaptic weight with an exponential dependence on the time delay between pre- and post-synaptic signals, mimic efficiently biological systems.

The possibility of developing and exploit photonic non-volatile synapses has been explored also by Chakraborty et al., who demonstrated that the proposed computing platform can be used to emulate a Spiking Neural Network inferencing engine for complex operations, such as image classification [121,122]. Wu et al. have recently demonstrated that a programmable waveguide mode converter based on GST225 can be programmed at 64 distinguishable levels, which are used to represent the weight parameters in matrix-vector multiplication computation with neuronal networks. They demonstrate the capability of the obtained system of performing image processing tasks, such as edge detection and pattern recognition, paving the way for the development of efficient optical computing [123].

## 6. Conclusions and Outlook

PCMs are a special subset of stimuli-responsive materials that offer a wide gamut of applications in optics and electronics. The rapid and drastic variation of their optical properties, in particular refractive index and extinction coefficient, opens the door to the burgeoning field of active photonics and optoelectronics. This work reviewed some relevant examples in the fields of optics, photonics and optoelectronics. In this context, VO_2_ and different types of GSTs are the most investigated materials and several architectures have been explored. A very important sector is represented by dynamic displays. GSTs thin films enable to fully exploit interference effects with other oxide layers that serve either as substrates (e.g., ITO) or thermal (e.g., Ta_2_O_5_) and diffusion (Ti/TiN) barriers. The fine-tuning of the degree of crystallization/amorphization of the PCMs is a key factor for the development of multicolored dynamic displays that can be controlled by direct or indirect (i.e., electrically- or light-driven) thermal stimuli. The use of PCM-based metasurfaces that take advantage of dynamic optical resonances is a very useful strategy for fabricating ON/OFF bicolor displays. VO_2_ thin films and metasurfaces represent a promising evolution of PCM-based displays that can operate either in IR or Visible. In particular, the research on rewritable metacanvas is not limited to displays but can be extended to the fabrication of optical devices embedding multiple functions, such as beam steering and hologram generation. In addition to dynamic optical displays, PCMs can offer a variety of applications in optical switching that is at the basis of reconfigurable photonic devices requiring either non-volatile (obtained by GSTs) or volatile (obtained by VO_2_) phase transitions. In particular, VO_2_ is suitable for low-power devices, asking for low-intensity optical modulation. PCMs have been extensively integrated into photonic waveguides and resonators, planar thin films and patterned photonic structures operating in different ranges of the electromagnetic spectrum, from Visible to Terahertz. PCMs can also be coupled to nanoantennas and nanoresonators, allowing us to control their optical resonances. Metamaterials and metasurfaces can take full advantage of such a very exciting opportunity, which could be transformed into a real breakthrough for active nanophotonics and sensing. Moreover, examples of the applications of PCMS in computing and information storage have been reviewed, showing the impressive progress in the development of photonic memory devices. Nucleation-dominated crystallization still represents a major bottleneck for phase-change memories, however huge advancement has been made in the last years, making PCM thin films attractive for large-scale production. In fact, the possibility to deposit PCM thin films at low temperature on flexibles substrates is promising for roll-to-roll fabrication of large-area devices. 

In addition to pioneering works on all-dielectric reconfigurable optical devices and metamaterials, many other fields of applications remain unexplored. In particular, ultrasensitive vibrational spectroscopies (e.g., Raman and IR) can be a profitable next frontier for all-dielectric PCMs, in view of leading the development of portable, ultra-compact sensors that overcome the low reproducibility and stability issues of current high-sensitivity devices [124,125,126,127,128]. Another broad field of possible applications for PCM-based thin films and nanostructures is nonlinear optics. While nonlinear optical effects in chalcogenides’ have been extensively investigated (see, e.g., [129]), little to no attention has been so far devoted to change of nonlinear optical interactions driven by the phase transition in PCM chalcogenides and VO_2_, such as modulation of harmonic generation and variations of self-phase modulation. 

Further promising fields of applications of PCMs encompass smart catalysts for environmental remediation [130,131,132,133] and system chemistry applied to solid-state devices [134]. Broadening the horizons of research could stimulate the discovery and development of new materials and architectures, with major, yet unpredictable benefits for different areas of application. Intense efforts and giant steps forward can be expected in the next years, as not only the “times”, but also “phases” need to rapidly change. 

## Data Availability

The data underlying this article will be shared on reasonable request from the corresponding author.
